# Supplementary material for: Cavity-enhanced optical readout and control of nuclear spin qubits
Source: Nat Commun. 2026 Aug 29;17:9234. doi: 10.1038/s41467-026-77060-8 (PMC13526025; doi:10.1038/s41467-026-77060-8)
Supplement: Supplementary file 1 [file 41467_2026_77060_MOESM1_ESM.pdf]

Supplementary information: Cavity-enhanced optical readout and control of nuclear spin qubits

Alexander Ulanowski, Johannes Früh, Fabian Salamon, Adrian Holzäpfel, and Andreas Reiserer*
Max-Planck-Institut für Quantenoptik, Quantum Networks Group,
Hans-Kopfermann-Straße 1, D-85748 Garching, Germany
Technical University of Munich, TUM School of Natural Sciences,
Physics Department and Munich Center for Quantum Science and Technology (MCQST),
James-Franck-Straße 1, D-85748 Garching, Germany and
TUM Center for Quantum Engineering (ZQE), Am Coulombwall 3A, 85748 Garching, Germany

I. TECHNICAL DETAILS OF THE EXPERIMENT

A. Cavity parameters

To predict the Purcell enhancement and the photon extraction efficiency, the longitudinal and transverse cavity modes were characterized in transmission for different mirror separations of the experiment cavity. Using the resonant frequencies, the mode geometry of the plano-concave resonator was fitted numerically, following the approach described in Ref. [1]. We extracted the radius of curvature of the depression, $ROC = 65(2)\,\mu\mathrm{m}$, a crystal thickness of $t_c = 10.90(2)\,\mu\mathrm{m}$, and a separation of the curved mirror from the crystal surface $t_a = 16.1(1)\,\mu\mathrm{m}$. The field decay rate of the resonator $\kappa = 2\pi \times 33(3)\,\mathrm{MHz}$ is determined from the linewidth of the fundamental (00) mode. This allows estimating the outcoupling efficiency on the side of the flat mirror for light polarized along the D2-axis of the crystal, $\kappa_{\mathrm{out}}/\kappa = 76(5)\,\%$. Including the fiber-coupling efficiency and other free-space losses, the total efficiency of extracting an intracavity photon into an optical fiber is around $60\,\%$.

The model also enables us to calculate the mode waist, $3.87(4)\,\mu\mathrm{m}$, and thus the coupling strength of a single emitter at the field maximum, $g_{\mathrm{max}} = 2\pi \times 127(3)\,\mathrm{kHz}$. Together with the polarization decay rate of Er:YSO, $\gamma = 2\pi \times 7\,\mathrm{Hz}$, we can predict the maximum Purcell enhancement as $P_{\mathrm{max}} = 140(8)$, in good agreement with experimental observations [2].

The second cavity, used for stabilization, was not characterized in detail. However, a similar cavity linewidth was observed, suggesting comparable cavity losses and outcoupling efficiency.

B. Cryostat setup

The experiments were performed in a ^{4}He pulse-tube based closed-cycle cryostat (see Methods section). To reduce fluctuations of the mirror separation induced by the pulse-tube, a custom-built vibration isolation setup (Fig. S1) was employed, which is largely based on the one described in Ref. [1]. However, the dampers between the sample stick and the main part of the cryostat, which were initially compressed air dampers, have been replaced by elastomeric dampers (NewPort M-ND01-A) to eliminate drifts in the sample position from air pressure fluctuations. Additionally, the metal bellows connecting the top plate to the sample space has been replaced by a rubber bellow to increase the damping.

C. Optical free-space setup

To couple both cavity modes to optical fibers, we use zoom collimators (Thorlabs ZC618APC-C). In between, the free-space beam paths are approximately 1.3 m long. A simplified schematic is shown in Fig. S1 (red and purple lines). Directly at the outcoupling mirror, an aspheric lens reduces the beam divergence. Four additional AR-coated lenses (focal length $f = 15\,\mathrm{cm}$) along the sample stick form a telescope to reduce the beam pointing sensitivity. Both beams leave the sample space through a window in the top plate and are deflected by 45°-mirrors into the horizontal plane. Using two adjustable mirrors (not shown), coupling efficiencies $> 95\,\%$ into anti-reflection-coated

* andreas.reiserer@tum.de

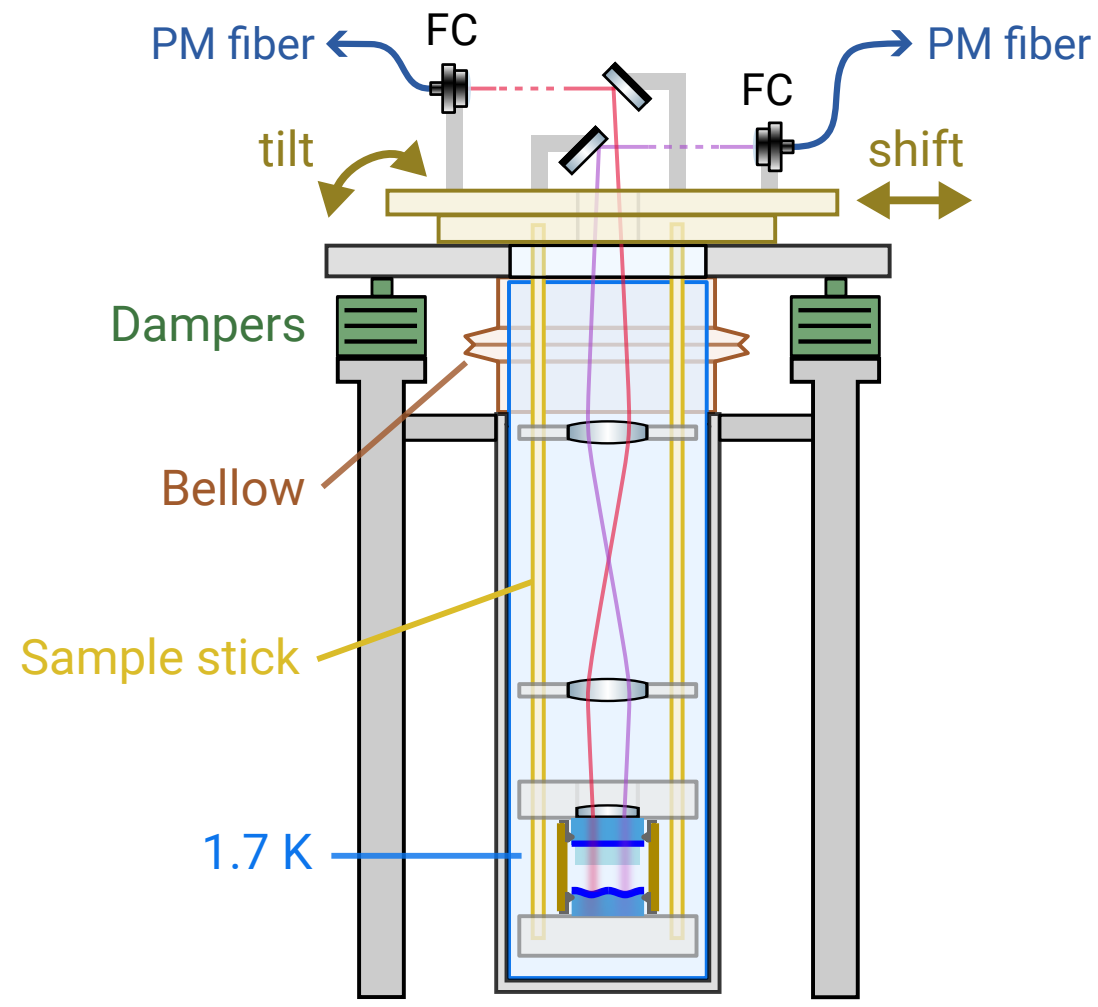

Supplementary Fig. S1. **Vibration isolation and optical free-space setup (not to scale).** Cross-section view of the cryostat sample space, including a simplified schematic of the two free-space beam paths between the optical cavities and the fiber collimators (FC). The sample stick with the cavity mount (bottom part) is vibration-isolated from the cryostat via passive dampers and a rubber bellow. Thermalization is achieved via Helium exchange gas (light blue). To avoid contact with the inner walls, the stick can be tilted and shifted within the sample space. Optical filters and waveplates on the top plate (yellow) are not shown.

polarization-maintaining (PM) fibers are reached. $\lambda/2$ and $\lambda/4$ waveplates allow adjusting the polarization (not shown). In addition, color filters prevent scattered light from the stabilization laser from reaching the single-photon detector. For this purpose, two long-pass filters (Semrock) with a cut-off wavelength at 1550 nm were placed into the stabilization path to suppress broadband amplified spontaneous emission of the laser at the erbium resonance frequency. A 2-nm-wide bandpass filter (Semrock) centered around 1535 nm in the spectroscopy path was used to block light at the wavelength of the stabilization laser.

D. Cavity tuning and stabilization

The mirror distance can be tuned via a piezo-electric tube (Physik Instrumente (PI) SE & Co. KG) up to a tenth of a free spectral range at cryogenic temperatures using voltages between -150 V and 150 V. The latter are generated by a low-noise high-voltage driver based on [3]. As illustrated in Fig. S2a, the mirror separation is actively stabilized to a laser reference using the Pound-Drever-Hall (PDH) technique and an open-source digital servo [4]. This allows for noise suppression up to a bandwidth of ≈ 0.6 kHz, which is limited by mechanical resonances of the cavity mount. Further software customizations to the servo software were implemented to enable automatic locking and monitoring of the cavity.

To avoid laser-induced heating in the part of the crystal used for optical spectroscopy, a different cavity structure, spatially separated by 0.42 mm, was chosen for length stabilization. To dynamically adjust the lock setpoint of the cavity resonances, a modulation signal with variable frequency $\nu_{\mathrm{L}}(t)$ is applied to the electro-optical modulator (EOM), which creates two symmetric first-order sidebands around the laser carrier frequency. For the PDH scheme, a secondary modulation signal at a constant frequency $\nu_{\mathrm{PDH}} < \nu_{\mathrm{L}}(t)/2$ is used to create an additional pair of sidebands around each tone. By tuning $\nu_{\mathrm{L}}(t)$, adjustments to the cavity resonance were performed on millisecond time scales within a range up to ≈ 1.5 GHz.

Vibration-induced electrical noise through the triboelectric effect [5] was minimized by choosing a flexible coaxial cable (Cryoandmore) for the connection of the piezo-electric actuator to the electrical feedthrough. However, at large magnetic fields > 1 T, additional electrical noise has been observed, leading to reduced stability of the mirrors that could not be fully compensated by the feedback loop. We attribute this effect to magnetically induced currents in open loops of the electrical wiring due to residual motion of the sample stick. This results in root-mean-square cavity frequency fluctuations of up to 23(7) MHz, which corresponds to approximately one third of the cavity linewidth (FWHM).

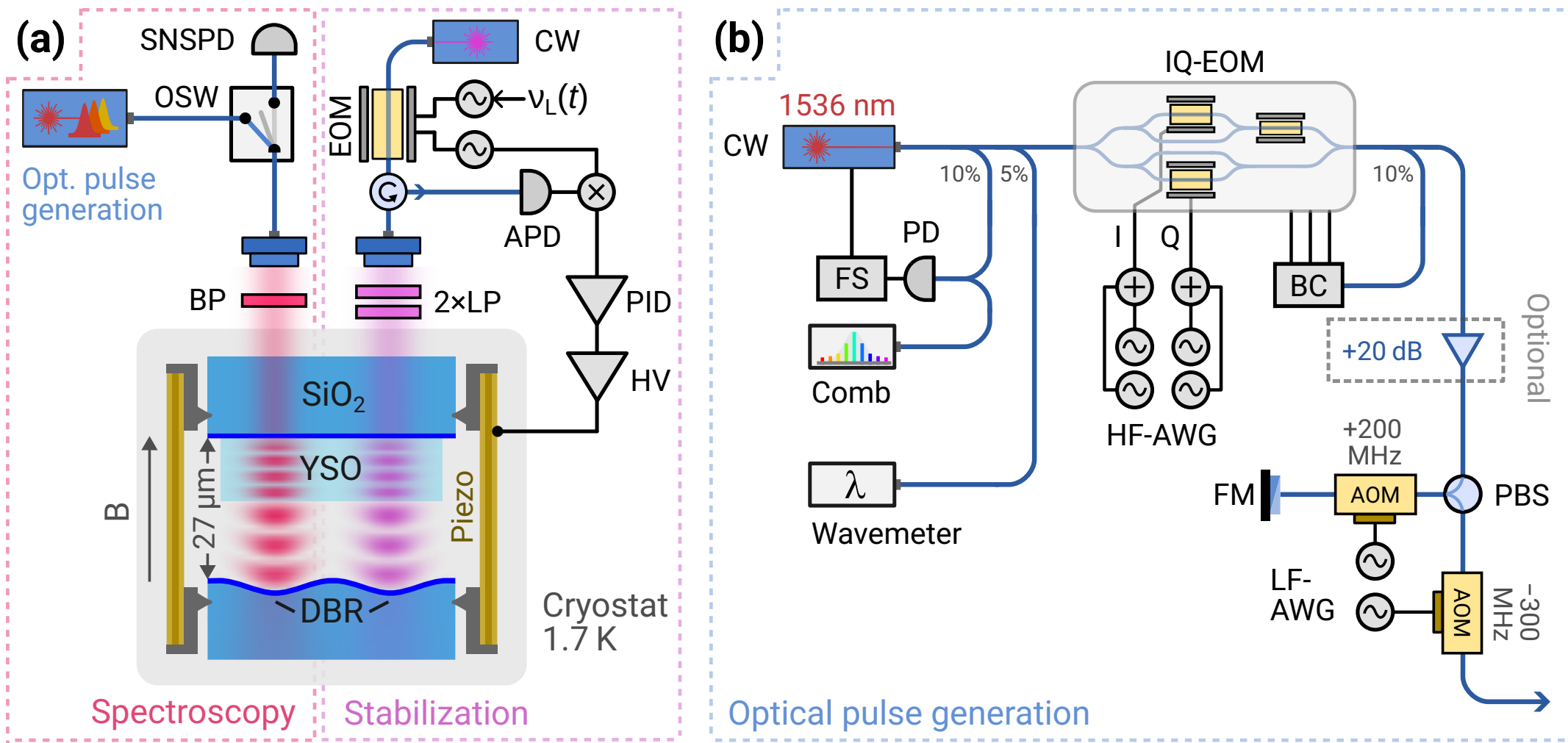

Supplementary Fig. S2. **Optical setup and electronics.** (a) Schematic of the optical Fabry-Perot resonators (not to scale) and experimental setup for optical spectroscopy and resonator stabilization. (b) Detailed schematic of the optical pulse generation setup. Optical PM fibers are shown in blue and electrical connections in black. Used abbreviations: AOM: acousto-optical modulator, APD: avalanche photodetector, AWG: arbitrary waveform generator, BC: bias controller, BP: band-pass filter, DBR: distributed Bragg reflector, EOM: electro-optical modulator, FM: Faraday mirror, FS: laser-frequency stabilization, HV: high voltage, HF: high-frequency, LF: low-frequency, LP: long-pass filter, OSW: optical switch, PBS: polarization beam splitter, PD: photodetector, SNSPD: superconducting nanowire single-photon detector.

E. Optical pulse generation and fluorescence detection

For optical excitation, a frequency-stabilized continuous-wave (CW) laser is used, which is modulated by several fiber-coupled acousto- and electro-optical modulators (AOMs and EOMs) to shape the optical pulses with an extinction ratio > 100 dB and durations < 1 µs. This setup is summarized in Fig. S2b and described in the following.

For spectroscopy, we use a fiber laser (NKT Photonics Koheras BASIK X15) with a free-running short-term linewidth of $\lesssim 1$ kHz. Tunable diode lasers (Toptica CTL or DLpro) are employed for stabilizing the Fabry-Perot cavity using the PDH technique, where high tunability is advantageous and stability requirements are less stringent. All lasers are frequency-stabilized via a phase lock to a frequency comb (Menlo Systems FC1500-250-ULN, 250 MHz repetition rate). The comb itself is stabilized to an optical reference cavity (Menlo Systems ORS1500), which enables achieving a linewidth of a few Hz for all used lasers. To this end, the beat between the laser and the comb is detected by Thorlabs PDA05CF2 photodetectors, and the resulting radio-frequency (RF) signal is mixed down to frequencies below 10 MHz. After amplification, this signal is used as the input to a digital phase-locked loop for active stabilization of the laser frequency. Phase detection and a PID filter are realized using the open-source digital servo described in Ref. [4]. Additionally, all lasers were referenced to a wavemeter (HighFinesse) with a resolution better than 40 MHz, which, together with the frequency comb, allows unambiguous determination of the absolute laser frequency.

The optical pulse generation setup consists of one IQ-EOM and two AOMs. In this configuration, the IQ-EOM (iXblue/exail MXIQER-LN-30) is used for optical single-sideband modulation with up to 30 dB extinction ratio, which is enabled by a bias controller (iXblue/exail MBC-IQ-LAB) that continuously monitors the optical output via a 90:10 fiber-based coupler. The I and Q inputs of the modulator are driven by a high-frequency arbitrary waveform generator (HF-AWG) from Zurich Instruments (model SHFSG). For two-frequency modulation, as required for stimulated Raman transitions, a low-frequency arbitrary waveform generator (LF-AWG) from Zurich Instruments (model HDAWG) is combined with the outputs of the HF-AWG.

Two fiber-coupled AOMs (Gooch & Housego) increase the extinction ratio (> 100 dB) and the dynamic range. Additionally, one of the AOMs is used to generate frequency-chirped pulses with a range up to 30 MHz. One of them creates a 200 MHz up-shift and is operated in a double-pass configuration using a Faraday mirror and a polarization beam splitter. The second AOM creates a 300 MHz down-shift in a single-pass configuration. Both AOMs are driven by the LF-AWG. All RF and MW outputs of the AWGs were amplified using TB-PMA3-83MP+ (Mini-Circuits) or AMPA-B-34 (AA Opto-electronic). In experiments that required a high laser power, an optical amplifier (Thorlabs BOA1004PXS) was inserted after the IQ-EOM and before the AOMs to compensate for their insertion losses.

Combined, the setup offers a high degree of flexibility for pulse sequences with amplitude and phase control, and fast frequency switching in the bands -1.4 .. 0 GHz and 0.2 .. 1.6 GHz relative to the carrier frequency of the laser,

making it suited for optical control of the nuclear spin of Er dopants.

For fluorescence detection, we use SNSPDs from PhotonSpot Inc., or ID Quantique SA, offering a high detection efficiency (up to 80 %) and low dark count rates. Adjusting the biasing current allows reducing the dark counts at the price of a reduced quantum efficiency. To switch the optical path to and from the cavity between the pulse generation setup and the detector, an optical switch (Agiltron NSSW-125225333-D050) is used, triggered by one of the AWGs. Single-photon detection events were recorded and processed using a time-to-digital converter (Swabian Instruments, Time Tagger 20).

II. HYPERFINE LEVELS IN SITE 1 OF ^{167}Er:YSO

A. Theoretical modeling

The hyperfine level structure of ^{167}Er:YSO is determined by the electric quadrupole and the magnetic dipole interaction, leading to a complex structure of the energy levels, as sketched in Fig. S3a. At low magnetic fields $\mathbf{B}$, it can be modeled by a reduced spin Hamiltonian, in which the electronic spin is treated effectively as $S = 1/2$ and no crystal-field Hamiltonian [6] is required. In this framework, the electronic Zeeman Hamiltonian is simply extended by the nuclear Zeeman term and the electron-nuclear interactions. The latter are described by two symmetric 3×3 tensors $\mathbf{A}$ and $\mathbf{Q}$ for the magnetic hyperfine and electric-quadrupole interactions, respectively. With the electronic and nuclear spin angular momentum operators $\mathbf{S}$ and $\mathbf{I}$, respectively, the resulting spin Hamiltonian reads [7]:

$$\mathcal{H}_{\mathrm{hf}} = \mu_{\mathrm{B}}\mathbf{B} \cdot \mathbf{g} \cdot \mathbf{S} - \mu_n g_n \mathbf{B} \cdot \mathbf{I} + \mathbf{I} \cdot \mathbf{A} \cdot \mathbf{S} + \mathbf{I} \cdot \mathbf{Q} \cdot \mathbf{I}. \tag{1}$$

Here, the nuclear g-factor of ^{167}Er is $g_n = -0.1611$ [8], and μ_{B} and μ_n denote the electronic and nuclear magnetons, respectively. Free parameters in the tensors $\mathbf{g}$, $\mathbf{A}$, and $\mathbf{Q}$ can either be fitted to experimental data or derived from crystal-field wave functions.

At large magnetic fields, however, the mixing of nearby crystal-field levels also affects the nuclear spin states. In this regime, the description via the reduced spin Hamiltonian in Eq. 1 gets inaccurate [9, 10]. In particular, the magnetic field sensitivity of the hyperfine transitions can be significantly enhanced beyond the contribution from the nuclear Zeeman effect alone. Therefore, a full modeling requires precise knowledge of the crystal-field Hamiltonian, which can be found in [11] and [6]. However, owing to the large number of fit parameters and the finite size of experimental datasets, extrapolating a crystal-field model to new parameter regimes may be inaccurate. This can be seen in Fig. S3b in a direct comparison of the mentioned models with experimental data (obtained using the techniques detailed in Sec. II B). While the models predict a nearly linear dependence of the level separation on the nuclear spin quantum number, the experimental data (colored dots) exhibit a significant curvature.

The reason is that only the magnetic hyperfine interaction and the $4f$-electron quadrupole contribution are included in the modeling. While the former results in a constant offset for all hyperfine ground (δ) and excited ($\bar{\delta}$) state splittings, the latter defines the linear slope. However, a convex shape, as observed in the data, can only be explained by a lattice contribution with complex-valued parameters [11]. This would also be consistent with the variation observed across different satellite lines (colors) in the inhomogeneous distribution that originate from europium co-dopants [2]. Still, a precise fit to extend the crystal field modeling would require additional experimental data under different magnetic field configurations, either for the same dopant or in the ensemble, which is left for future work.

While the experimental techniques to obtain the transition frequencies will be explained in more detail in Sec. II B, in the following, we will first discuss the optical spectra, as the respective transition frequencies determine whether the optical readout of nuclear spin qubits is achievable. Against this backdrop, Fig. S4a shows the magnetic-field dependence of the optical transition frequencies (solid lines), calculated using the crystal-field model for a magnetic field applied along the b-axis of YSO. For comparison, also the prediction from the effective spin-1/2 Hamiltonian is shown as dotted lines, which increasingly deviate at magnetic fields $B \gtrsim 0.5\,\mathrm{T}$. Still, in both models, the relative frequency detuning between the spin-preserving and spin-flip transitions remains mostly unchanged when a magnetic field up to $10\,\mathrm{T}$ is applied. Thus, low-field measurements may already be sufficient to extrapolate whether the linewidth of a given resonator is much smaller than the splitting of the spin-flip hyperfine transitions, such that optical nuclear spin readout becomes feasible. Comparing the predicted transition frequencies to the experimental values gives qualitative agreement, with larger deviations for the $\Delta m_I = +1$ transitions.

To determine the best transition for the optical readout, it is furthermore instructive to look at the branching ratios of the optical transitions. In a first approximation, this can be derived from the effective electronic spin-1/2 Hamiltonian [11] at moderate B-fields of $B = 0.5\,\mathrm{T}$ along the b-axis. The result is shown in Fig. S4b. As can be seen, the branching ratio is highest for the states $|-7/2\rangle$ and $|+7/2\rangle$. Thus, our experimental implementation focused on these transitions.

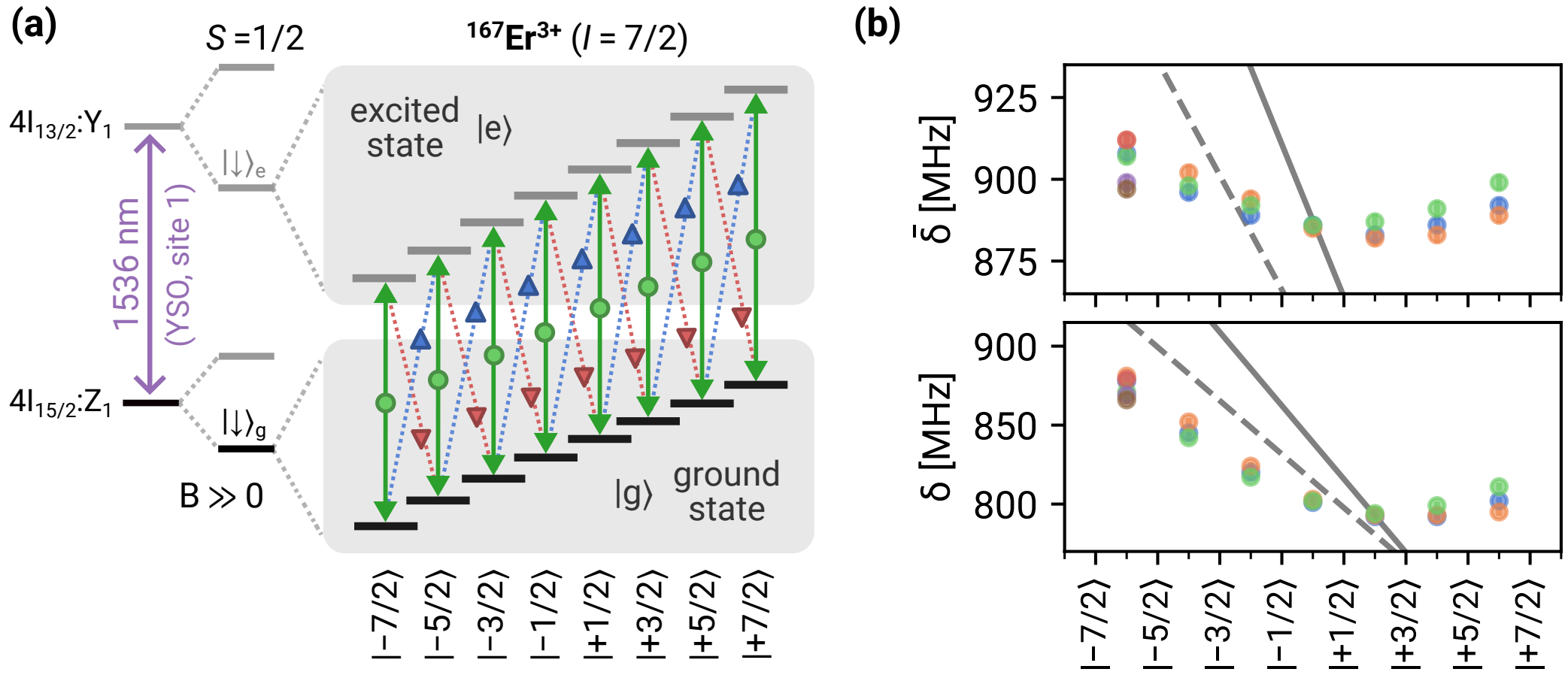

Supplementary Fig. S3. (a) Hyperfine level structure in the ground (Z_1) and the optically excited state (Y_1) when the electronic spin is frozen in $|\downarrow\rangle_g$. The dominant optical transitions are either nuclear-spin preserving (solid green, $\Delta m_I = 0$) or flip the spin by $\Delta m_I = -1$ (dotted red) or by $\Delta m_I = +1$ (dotted blue). (b) Measured hyperfine splittings in the ground (δ, bottom panel) and the optically excited ($\bar{\delta}$, top panel) states of ^{167}Er in different satellite lines (colored dots) of site 1 when a magnetic field of $B = 6.8\,\mathrm{T}$ is applied along the b-axis of the crystal. The solid and dashed lines show a crystal-field prediction of the hyperfine splittings, derived from parameters reported in [11] and [6], respectively.

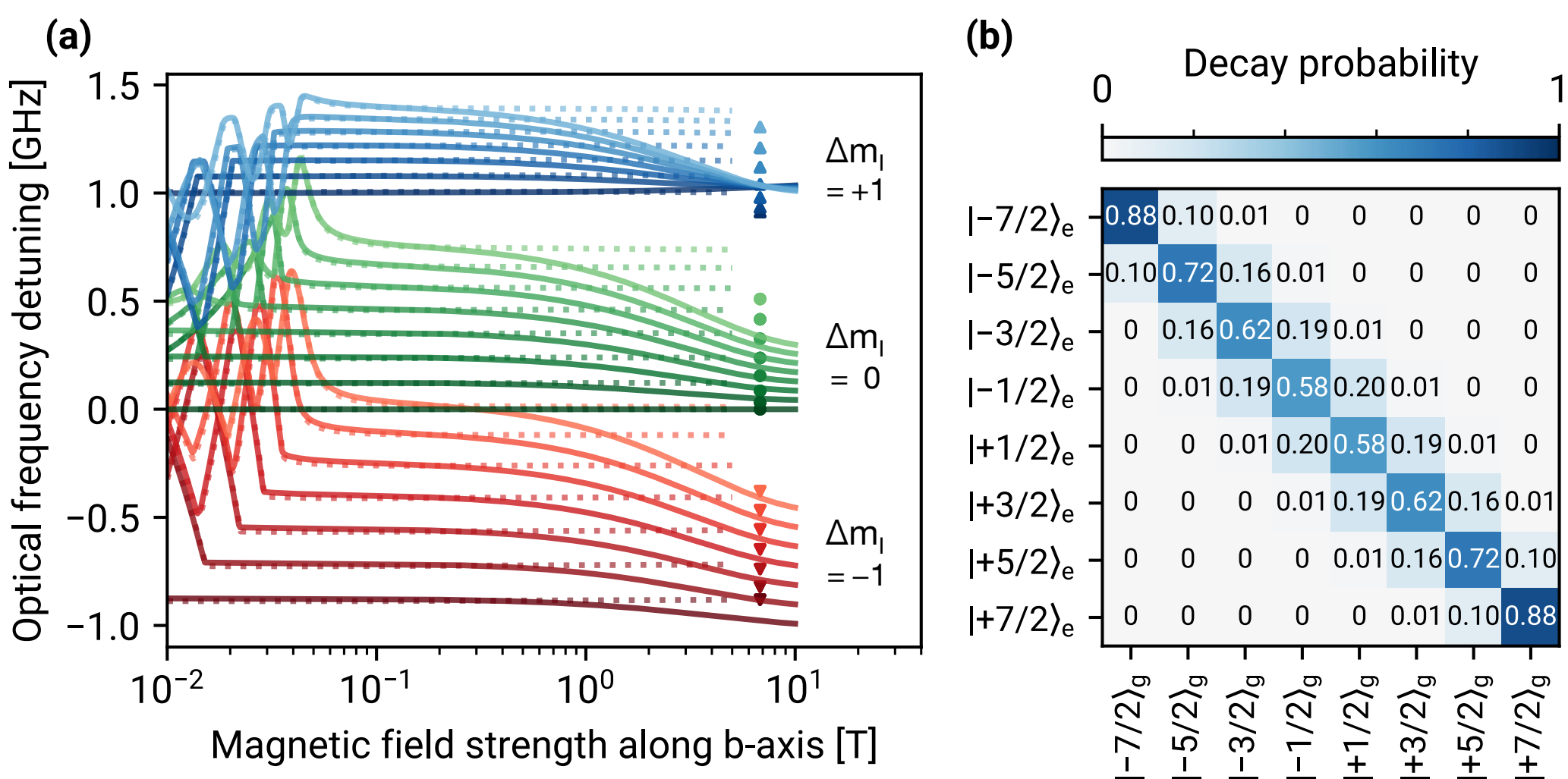

Supplementary Fig. S4. **Optical transitions and branching ratios of ^{167}Er dopants (site 1) in YSO.** (a) Optical frequency detuning of the $\Delta m_I = 0, \pm 1$ transitions relative to the lowest spin-preserving transition $|-7/2\rangle_g \leftrightarrow |-7/2\rangle_e$, as a function of the magnetic field strength along the b-axis of the crystal. Solid lines show the prediction of a crystal-field model [11], while the dotted lines are derived from a reduced spin Hamiltonian with an effective electronic spin-1/2. Considerable deviations are observed for magnetic fields $B \gtrsim 0.5\,\mathrm{T}$, due to the mixing of adjacent crystal-field levels. Experimental data obtained at $B = 6.8\,\mathrm{T}$ is shown with circle and triangle markers. (b) Branching ratios of the optical transitions derived from the effective electronic spin-1/2 Hamiltonian [11] at $B = 0.5\,\mathrm{T}$, along the b-axis.

At large magnetic fields, the crystal-field model shows similarities to the experimental data, indicated by the circle and triangle symbols in Fig. S4a. In particular, the spin-preserving transitions ($\Delta m_I = 0$) and spin-flip transitions ($\Delta m_I = \pm 1$) form "bands" that are separated by several hundred MHz, which eases optical spin pumping. Furthermore, as the minimum separation between adjacent transitions is on the order of 20 MHz—two orders of magnitude larger than the spectral diffusion linewidth—off-resonant driving of adjacent transitions can be easily avoided, facilitating single-shot readout.

B. Optical detection of single ^{167}Er dopants

In the following, we will describe the techniques used to determine the optical transitions of individual ^{167}Er dopants in more detail. To spectrally resolve single ^{167}Er dopants, their optical transitions should be clearly separated from those of other dopants. Thus, all experiments are performed on the satellite lines that result from europium co-dopants [2]. We then use two different experimental sequences to record fluorescence spectra—one without and one with optical spin pumping.

1. Fluorescence spectrum without optical spin pumping

In thermal equilibrium, the nuclear spin of ^{167}Er dopants occupies any of the eight hyperfine states $m_I = -7/2, ..., +7/2$ with nearly equal probability, since the thermal energy at the cryostat base temperature ($k_{\mathrm{B}}T \approx 35\,\mathrm{GHz}$) exceeds the total hyperfine splitting ($\approx 6\,\mathrm{GHz}$) under the experimental conditions of $B = 6.8\,\mathrm{T}$ applied along the b-axis of the crystal. As a result, the average intensity in resonance fluorescence measurements is expected to be $\approx 1/8$ of that obtained for dopants without hyperfine structure ($I = 0$). The fluorescence intensity may decrease further if the spin is optically pumped into a long-lived state where it is no longer resonantly excited. To avoid this, the temperature can be increased to reduce the hyperfine lifetime. Alternatively, all eight spin-preserving transitions can be addressed sequentially.

Since the cavity linewidth is much narrower than the typical optical splitting between transitions, its resonance is adjusted to match the excitation frequency of the optical pulses. With this, we record a fluorescence spectrum over a frequency range of 1.4 GHz, covering one magnetic class of the outermost red-detuned satellite line [2]. For optical excitation, we use laser pulses with a duration of 8 µs that are frequency-chirped over 2 MHz. We then record the photons emitted within 0.5 ms after the pulses are turned off. To avoid optical spin pumping into non-resonant states during the acquisition of the main spectrum, the frequency of the optical excitation pulses is increased in steps of 2 MHz after every 20th pulse. Simultaneously, the cavity resonance with a full-width-at-half-maximum (FWHM) of 65 MHz is adjusted in steps of 50 MHz to remain near-resonant with the excitation frequency. The result after averaging 45×10^3 repetitions at each frequency step is shown in Fig. S5 (dark blue trace).

The spectrum exhibits many well-resolved, narrow peaks, each of which originates from an individual Er dopant. Some of these peaks exhibit a short lifetime, which suggests a strong Purcell enhancement, but a reduced amplitude that is consistent with the eightfold reduction expected for ^{167}Er dopants in a random nuclear spin state.

In this particular spectrum, two spectrally close ^{167}Er dopants have been identified in the frequency range between -100 MHz and $+500$ MHz, relative to the lowest spin-preserving transition of one of the dopants. Using the method described later in Sec. II C, the full hyperfine structure of one dopant has been resolved, and the corresponding optical transitions are marked with circle and triangle symbols at the bottom of Fig. S5.

While the spectrum shown in Fig. S5 already indicates the presence of ^{167}Er dopants, it is not yet possible to assign individual peaks to specific optical transitions. Moreover, dopants with overlapping optical transitions cannot be distinguished, particularly near the center of the satellite line. This limitation is addressed in the following section through optical initialization of the nuclear spin.

2. Fluorescence spectrum with optical pumping

Initialization of the nuclear spin can be achieved via optical pumping on the spin-flip transitions [10], using either $\Delta m_I = +1$ or -1. In the scheme depicted in Fig. S6a, the target state is $|-7/2\rangle_g$. Thus, the dopant, initially in any of the hyperfine ground states, is optically excited via the red-detuned spin-flip transitions that decrease the nuclear spin quantum number. Due to the unequal optical branching ratios (see Fig. S4b), spontaneous relaxation to the ground state preferentially occurs via emission of a photon on the spin-preserving transition. The decay rate may be additionally enhanced by coupling to the optical cavity.

However, as a decay via other transitions cannot be excluded, the spin pumping is inherently probabilistic. We thus define a pump cycle as a series of optical pulses, applied sequentially to each of the seven $\Delta m_I = -1$ transitions. This cycle is repeated 500 times to maximize the chance of a successful spin transfer to the target state. After the last pump cycle, a waiting period of 20 ms is added before probing the fluorescence in order to minimize background fluorescence from unintentionally excited dopants. Then, as shown in Fig. S6b, we apply 250 probe cycles before repeating the initialization procedure, with 100 repetitions in total.

To achieve the most efficient pumping, one would need to precisely know all optical transition frequencies. However, as described in Sec. II C, their modeling may be inaccurate. Still, as the optical transitions with the same Δm_I are organized in spectral "bands" (see Fig. S4a), initialization in $|-7/2\rangle_g$ is possible even without precise knowledge of

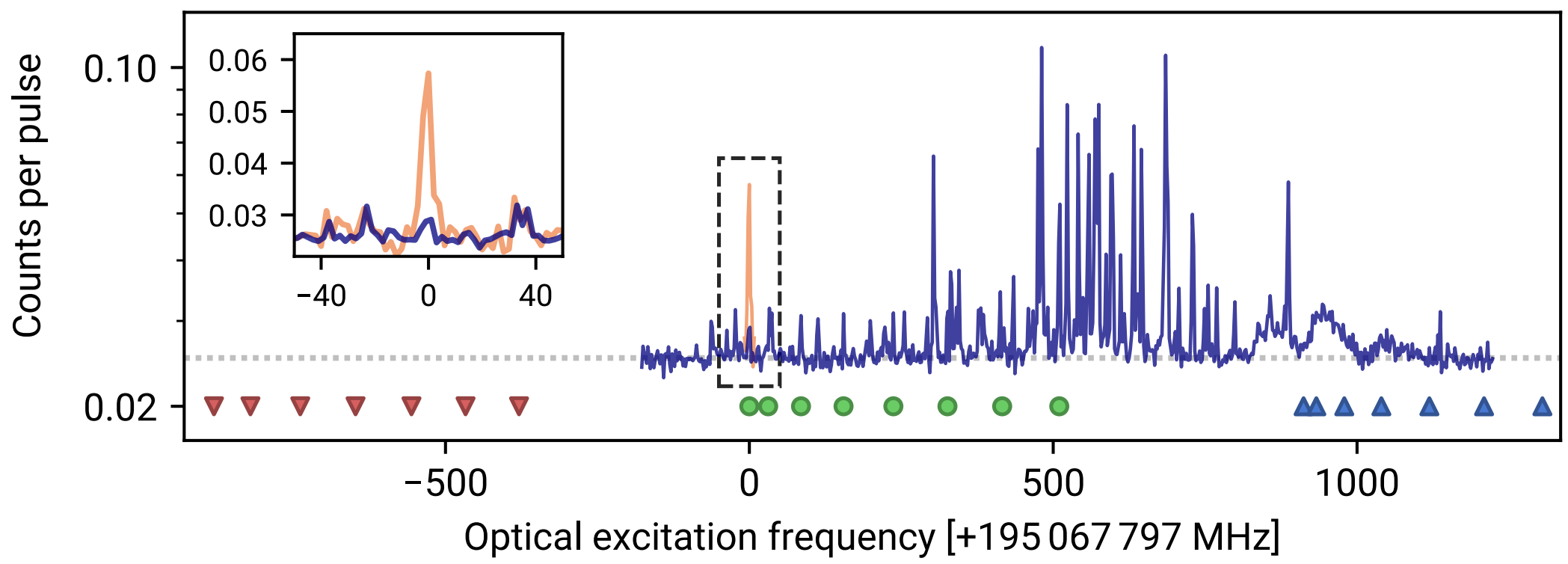

Supplementary Fig. S5. **Resonance fluorescence spectrum of individual Er dopants with and without hyperfine structure.** The spectrum shows the outermost red-detuned satellite line in an Eu co-doped YSO sample [2] at a magnetic field of $B = 6.8\,\mathrm{T}$ applied along the b-axis. The gray dotted line indicates the dark count level of the used detector. Prominent spectral features with high amplitude (up to 0.08 counts per pulse) originate from Er dopants without hyperfine structure ($I = 0$). In contrast, peaks with short lifetime, but approximately eightfold lower amplitude, can be attributed to the spin-preserving transitions of ^{167}Er dopants. Optical pumping of the $\Delta m_I = -1$ spin-flip transitions initializes the nuclear spin into $|-7/2\rangle_g$, resulting in increased fluorescence on the corresponding spin-preserving transition (orange trace). With similar measurements (not shown), all optical transitions of a single ^{167}Er dopant can be identified and are marked with circles ($\Delta m_I = 0$), red ($\Delta m_I = -1$), and blue triangles ($\Delta m_I = +1$).

all transition frequencies. To this end, we simply apply a frequency-swept optical pulse sequence addressing one of the three bands. As shown in Fig. S6c, for optical pumping (red), we chose a frequency window from $-0.2\,\mathrm{GHz}$ to $-1\,\mathrm{GHz}$ relative to the center of the probe window (green). For technical reasons, each pump cycle consists of a series of optical pulses with a duration of $20\,\mu\mathrm{s}$, each frequency-chirped over a range of $10\,\mathrm{MHz}$. After each optical pulse and a time delay of $20\,\mu\mathrm{s}$, the carrier frequency is shifted by $-10\,\mathrm{MHz}$, repeating this process until the entire pump window is covered. To compensate for the cavity detuning-dependent attenuation, the optical power of the pulses is adjusted accordingly.

After the last pump cycle, the fluorescence spectrum is probed using $2\,\mathrm{MHz}$-chirped pulses over a $100\,\mathrm{MHz}$ frequency window, which lies within the resonator bandwidth. Within this range, all ^{167}Er dopants are expected to be found in the $|-7/2\rangle_g$ state with high probability and thus exhibit strong fluorescence on the corresponding spin-preserving transition. However, with each of the 250 probe cycles, the nuclear spin can flip into a different state, resulting in a gradually decreasing fluorescence intensity. This can be seen in the resulting spectra, Fig. S6d, in which we average only over the first 30 (orange) or the last 30 (blue) probe cycles. Some peaks appear only in the orange trace, which can thus be attributed to ^{167}Er dopants. The remaining peaks that do not change in intensity originate from Er dopants without hyperfine structure.

C. Precise characterization of the hyperfine level structure

In previous studies, the hyperfine structure of an ^{167}Er ensemble in a bulk YSO crystal has been characterized optically via spectral hole-burning [10]. In this section, instead, we describe our experiments in which the hyperfine levels of individual ^{167}Er dopants are characterized. This allows for a high frequency resolution, and for observing differences between dopants caused by inhomogeneous effects, such as local strain induced by nearby co-dopants.

We start by selecting a fluorescence peak that corresponds to the $|-7/2\rangle_g \leftrightarrow |-7/2\rangle_e$ transition of a single ^{167}Er dopant from the spectrum in Fig. S6d. To determine the frequency splittings to the next higher-lying spin states, two sequences, denoted as "A" and "B", are iteratively applied to determine the frequencies of the corresponding optical transitions. This process is repeated until the $|+7/2\rangle_g$ state is reached. The sequences A and B are illustrated in Fig. S7 and are described in the following.

Sequence A: The ^{167}Er dopant is first initialized by optical pumping into a specific hyperfine ground state, denoted by $|i\rangle_g$ in Fig. S7a. Then, to determine the frequency of the spin-flip transition $|i\rangle_g \leftrightarrow |i+1\rangle_e$ originating from that state (blue dashed), a series of identical pump pulses is applied with varying detuning. When the pulses are on resonance with the transition, the dopant will be pumped to a different ground state, leading to a dip in the fluorescence signal ("spectral hole") when probing the optical transition from $|i\rangle_g$ (green).

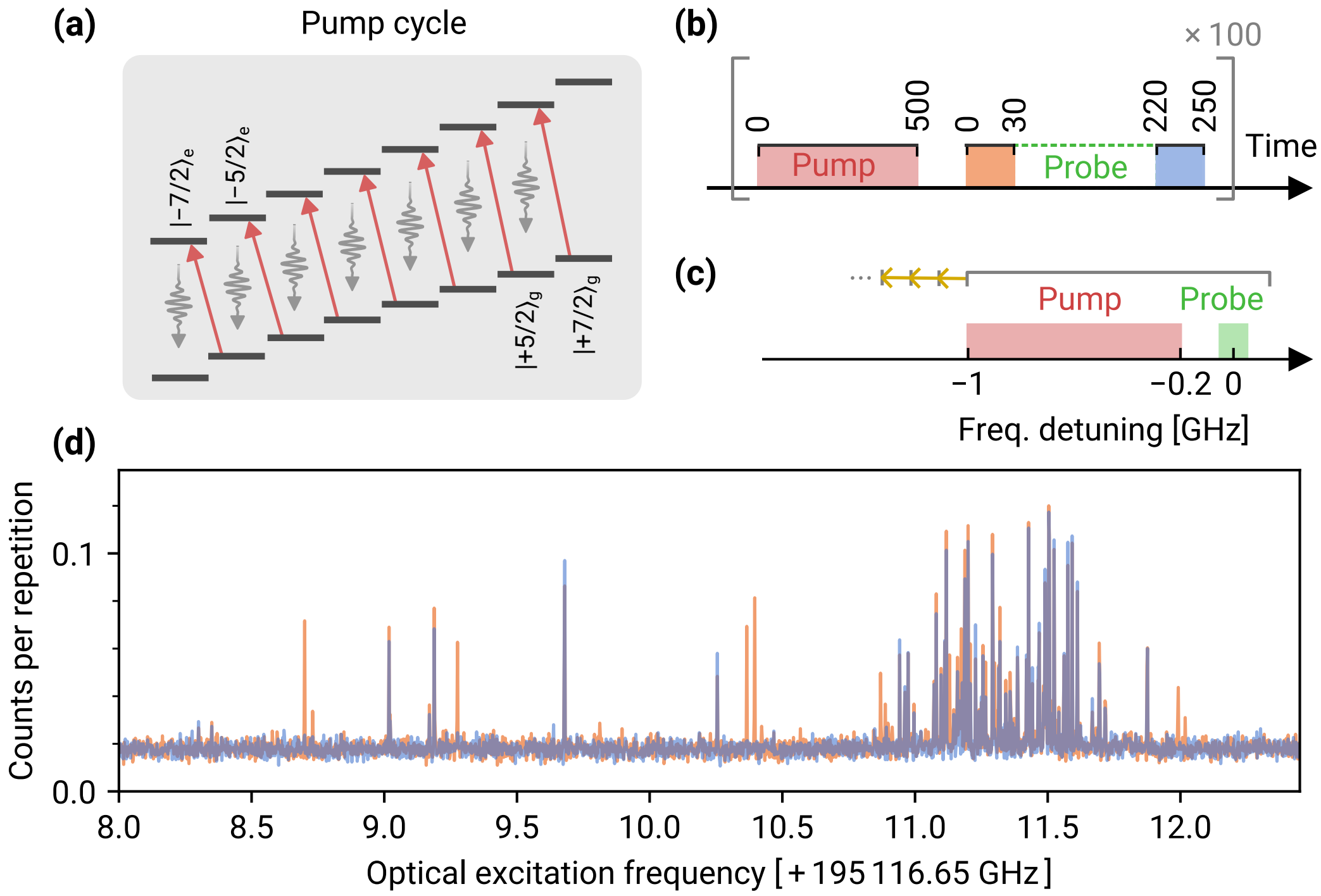

Supplementary Fig. S6. **Resonance fluorescence spectrum with optically initialized ^{167}Er dopants.** (a) Optical pumping scheme to transfer the spin population from thermal equilibrium to $|-7/2\rangle_g$. In one pump cycle, each of the red-detuned spin-flip transitions ($\Delta m_I = -1$, red arrows) is optically excited, while spontaneous emission (gray arrows) occurs predominantly via the spin-preserving transitions. Still, to increase the probability of a successful population transfer, this cycle is repeated 500 times. (b) Measurement sequence to acquire a fluorescence spectrum with spin initialization. Following the optical pumping described in (a), the fluorescence is probed in a frequency window of 100 MHz, in total using 250 probe cycles. In each probe window, the fluorescence is measured with a frequency resolution of 2 MHz using frequency-chirped optical pulses. The entire sequence is repeated 100 times. (c) Pump and probe cycles in the frequency domain. To acquire a wide fluorescence spectrum, after the last repetition of the measurement sequence in (b), the frequency detuning of the pump and probe windows is shifted by −100 MHz to acquire the next partial spectrum. (d) Fluorescence spectrum acquired in the first 30 (orange) and the last 30 (blue) probe cycles. Peaks appearing only in the orange trace are attributed to different ^{167}Er dopants in the $|-7/2\rangle_g$ nuclear spin state. After repeated optical probing, these dopants are pumped into a different spin state, which results in the reduced fluorescence in the blue trace.

Sequence B: The dopant is again initialized in the state $|i\rangle_g$, followed by optical pumping on the transition $|i\rangle_g \leftrightarrow |i+1\rangle_e$ determined by sequence A. This leads to a transfer of the population into the $|i+1\rangle_g$ state with a high probability. To determine the corresponding spin-preserving transition frequency $|i+1\rangle_g \leftrightarrow |i+1\rangle_e$, another fluorescence spectrum is recorded, this time as a function of the probe detuning (green). Additionally, a reference spectrum is acquired under the same conditions but without applying the optical pump pulses. Subtracting this reference spectrum from the one obtained with optical pumping reveals a peak (”spectral anti-hole”) at the spin-preserving transition.

Using the optical frequencies of the spectral hole and anti-hole, the splittings between adjacent hyperfine levels are determined as follows:

$$\delta_{|i\rangle}^{|i+1\rangle} = \Delta_{|i\rangle}^{|i+1\rangle} - \Delta_{|i+1\rangle}^{|i+1\rangle}, \tag{2}$$

$$\bar{\delta}_{|i\rangle}^{|i+1\rangle} = \Delta_{|i\rangle}^{|i+1\rangle} - \Delta_{|i\rangle}^{|i\rangle}, \tag{3}$$

where $\delta_{|i\rangle}^{|i+1\rangle}$ and $\bar{\delta}_{|i\rangle}^{|i+1\rangle}$ denote the hyperfine splittings in the ground and excited states, respectively, and $\Delta_{|i\rangle}^{|j\rangle}$ the measured frequency of the optical transition $|i\rangle_g \leftrightarrow |j\rangle_e$. Once the level spacings are known, the optical frequency of the red-detuned spin-flip transition is calculated using:

$$\Delta_{|i+1\rangle}^{|i\rangle} = \Delta_{|i\rangle}^{|i\rangle} - \delta_{|i\rangle}^{|i+1\rangle}. \tag{4}$$

To initialize the next ground state to be characterized, $|i+1\rangle_g$, the optical initialization sequence is modified by

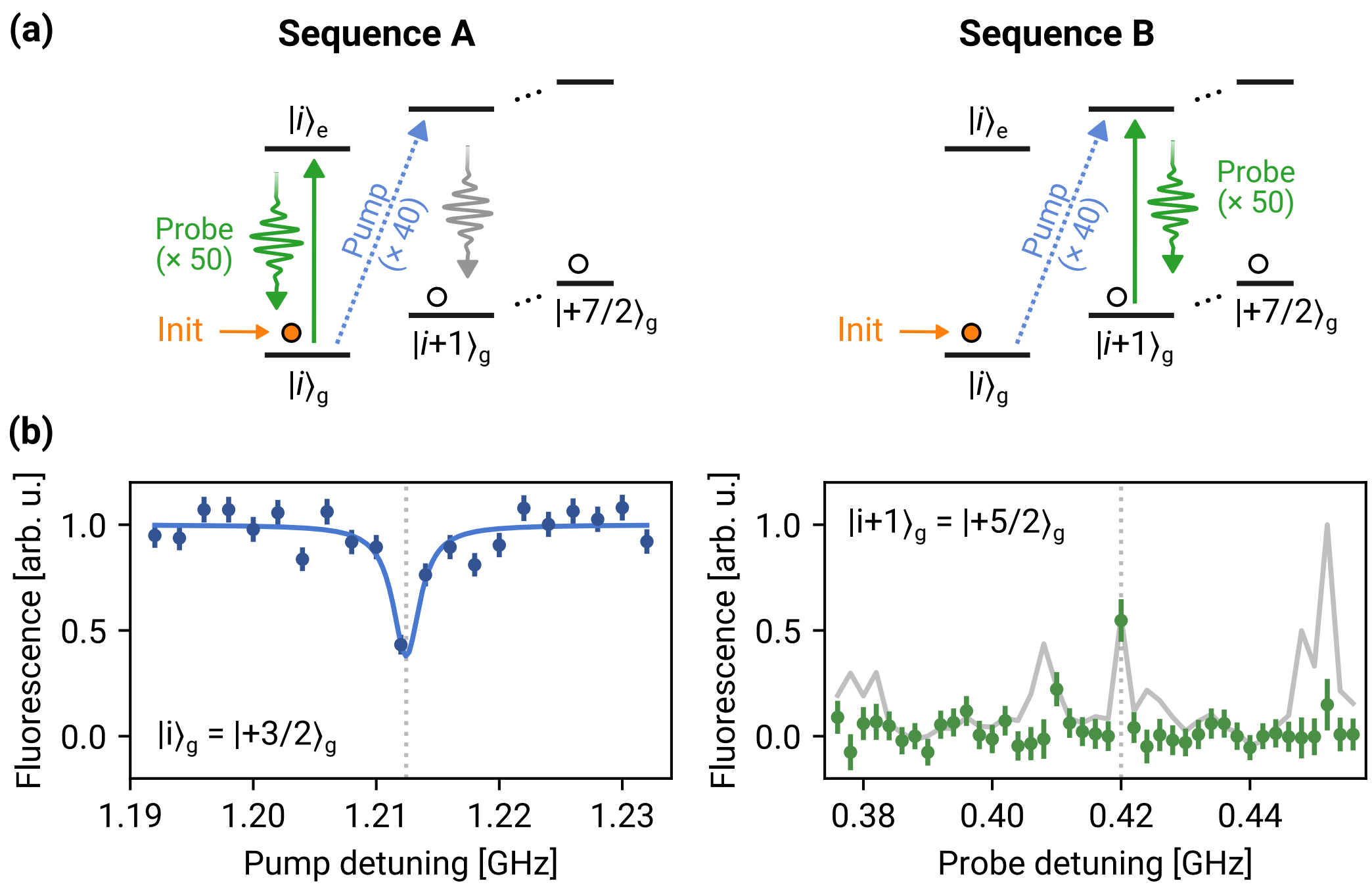

Supplementary Fig. S7. **Optical characterization of the hyperfine level structure.** (a) Two pump-probe sequences, labeled "A" and "B", which are used to determine the energy splittings within the four-level system $|i\rangle_{(g,e)}$ and $|i+1\rangle_{(g,e)}$, forming part of the larger $I = 7/2$ hyperfine manifold. To characterize the entire manifold, this scheme is iteratively applied to successive level pairs ($|i+1\rangle_{(g,e)}$ and $|i+2\rangle_{(g,e)}$). Further details are given in the main text. (b) Left panel: Fluorescence as a function of the pump detuning showing a dip that yields the frequency of the transition $|i\rangle_g \leftrightarrow |i+1\rangle_e$. Right panel: The background-subtracted fluorescence spectrum (green dots) after applying 40 pump pulses at $|i\rangle_g \rightarrow |i+1\rangle_e$ yields the frequency of the $|i+1\rangle_g \leftrightarrow |i+1\rangle_e$ transition, whereas the uncorrected spectrum (solid gray line) also contains spectral features attributed to Er dopants without hyperfine structure ($I = 0$). In both panels, fluorescence is probed using 50 frequency-chirped pulses over a range of 2 MHz. Error bars: 1 SD.

excluding the red-detuned spin-flip transition $\Delta^{|i\rangle}_{|i+1\rangle}$ from the optical pumping sequence, and instead adding the newly obtained blue-detuned spin-flip transition $\Delta^{|i+1\rangle}_{|i\rangle}$. Sequences A and B are then repeated for the next pair of hyperfine states: $|i+1\rangle_{(g,e)}$ and $|i+2\rangle_{(g,e)}$ until all levels are determined. Given the frequency-chirp range of 2 MHz used for both pump and probe pulses, the uncertainty in the extracted hyperfine splittings is estimated to be 2 MHz.

Fig. S3b shows the result of characterizing the hyperfine splittings of several dopants at a magnetic field of $B = 6.8\,\mathrm{T}$. Small variations between the dopants ($\mathcal{O}(10\,\mathrm{MHz})$) are attributed to inhomogeneous strain in the crystal.

D. Hyperfine lifetime

To measure the hyperfine lifetime, the nuclear spin is first initialized into the $|-7/2\rangle_g$ state and is probed on the corresponding SP transition after a variable waiting time τ_w, as indicated in the sequence diagram in Fig. S8a. The normalized, background-corrected fluorescence at a magnetic field of $B = 6.8\,\mathrm{T}$ along the b-axis of the crystal and at a sample temperature of 1.72(1) K is shown in Fig. S8b. A fit to an exponential decay yields a hyperfine lifetime of 33(3) s and an offset of 0.16(3), close to the expected value of 0.1353(2) in thermal equilibrium, derived from the ground-state hyperfine level structure and the sample temperature. At elevated temperatures, the hyperfine lifetime decreases, as shown in the inset of Fig. S8b. For temperatures above 1.72 K (red), no direct temperature measurement near the sample was possible; thus, the estimated uncertainty is included in the error bars.

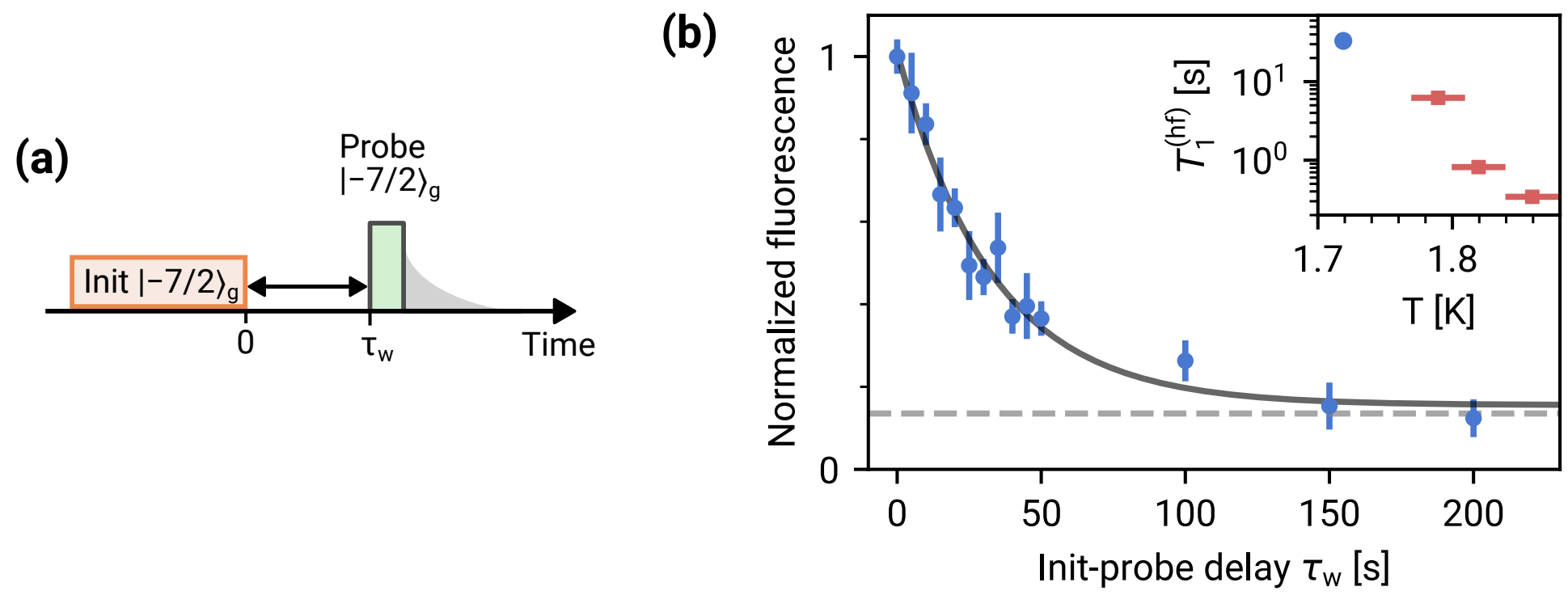

Supplementary Fig. S8. **Hyperfine lifetime of a single ^{167}Er dopant.** (a) Measurement sequence. The nuclear spin is first optically initialized into the $|-7/2\rangle_g$ state, and after a variable waiting time τ_w, the fluorescence is probed on the $|-7/2\rangle_g \leftrightarrow |-7/2\rangle_e$ SP transition. (b) Main panel: Normalized and background-corrected fluorescence as a function of τ_w. At a temperature of 1.72(1) K and a magnetic field of $B = 6.8\,\mathrm{T}$ (along the b-axis), a hyperfine spin lifetime of 33(3) s and an offset of 0.16(3) are extracted from an exponential fit (solid line). For long waiting times, the fluorescence converges to the expected intensity in thermal equilibrium (0.1353(2), dashed line). Inset: Temperature dependence of the hyperfine lifetime. Error bars: 1 SD.

III. OPTIMIZED OPTICAL INITIALIZATION OF THE NUCLEAR SPIN

Once the optical spin-flip transitions ($\Delta m_I = \pm 1$) have been characterized, the spin initialization scheme can be optimized. Instead of optically pumping a broad frequency range, each of the seven spin-flip transitions is addressed individually using narrowband pulses that are frequency-chirped over 10 MHz. A block consisting of these seven distinct pulses, which are separated by a waiting time $\tau_i = 0.2\,\mathrm{ms}$, is then defined as one initialization cycle in the optimized sequence, used throughout the main manuscript. Note that the waiting time is small compared to the optical lifetime of 11.4 ms in free space and for the high-lying nuclear spin states that are detuned from the cavity. Thus, many repetitions N_{init} of the initialization cycle will be required to obtain a high fidelity, defined as the probability of successfully preparing the target spin state.

To maximize this value while minimizing the initialization time, the optimal number of cycles is determined experimentally. To this end, we first prepare the nuclear spin in a known initial state distinct from the target state $|-7/2\rangle_g$ using 500 cycles. We then apply a variable number of initialization cycles and measure the population in the target state via fluorescence detection on the lowest spin-preserving transition, $|-7/2\rangle_g \leftrightarrow |-7/2\rangle_e$. The resulting population in $|-7/2\rangle_g$ for each of the initial states is shown as a function of N_{init} in Fig. S9b. The curves saturate at a value of 0.973(9) (see Sec. V D). In the least favorable case, where the spin is initially in the $|+7/2\rangle_g$ state, an initialization duration of at least 1 s is required to reach the saturation value. This duration could potentially be further reduced by optimizing the pump pulse sequence, e.g., by tuning the resonator to enhance the $\Delta m_I = 0$ or $+1$ transitions during the initialization procedure, or by introducing feedback to interrupt the pump sequence once the target state is reached.

A. Population dynamics during optical spin pumping

The experimental data in Fig. S9b, showing the transferred population as a function of N_{init}, can also be used to estimate the excitation probability on the spin-flip transitions. To model the spin dynamics during the initialization sequence, we consider the 16 hyperfine states (eight each in the ground and optically excited states), and the possible transitions between those, as depicted in Fig. S9a. Transitions between these states after a single initialization cycle are indicated by red and gray arrows, together with the respective transition probability. In this model, an optical pulse resonant with a spin-flip transition (red arrow) will swap the population of the respective ground and excited states with the probability p_i, otherwise leaving the population unchanged. The probability for a spontaneous relaxation from the excited to the ground state in between two optical pulses depends on the optical lifetime of the respective transition. For the chosen ^{167}Er dopant, the Purcell-enhanced lifetime varies between 0.6 ms and 11.4 ms, depending on the detuning to the cavity resonance. The branching ratios of the allowed transitions to the ground state, including

both spin-preserving and spin-flip, are derived from the spin Hamiltonian and are corrected for cavity enhancement. Due to the long lifetime of the nuclear spin exceeding 30 s, state transitions within the ground and excited states are not considered.

A fit based on the described model with p_i as free parameters is shown using solid lines for different initial states in Fig. S9b. The extracted values of the transition probabilities p_i (inset) show a decrease with increasing spin quantum number due to the larger detuning to the cavity resonance of the respective transitions. However, p_3 exhibits an abnormally high value compared to all other transition probabilities, which could originate from higher-order resonant cavity modes.

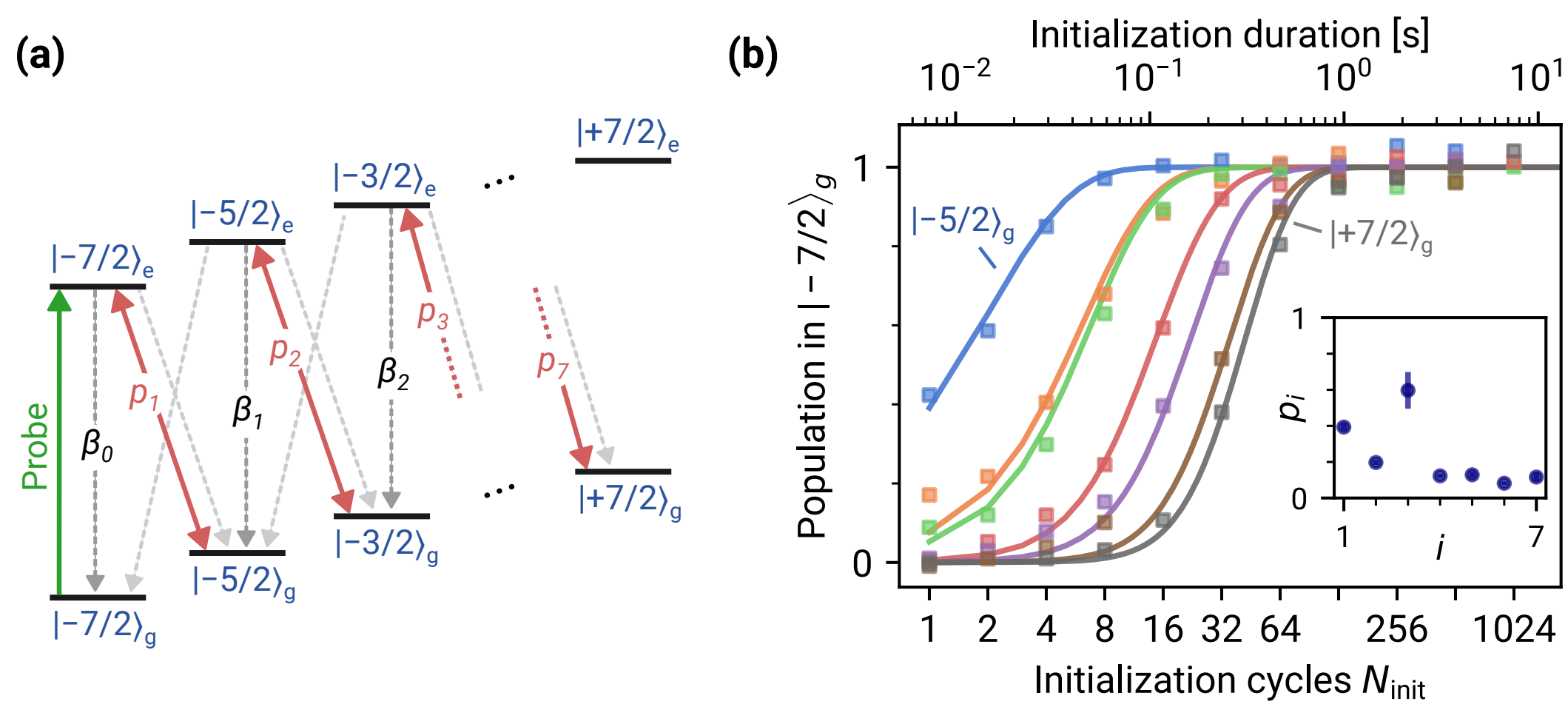

Supplementary Fig. S9. **Optical nuclear spin initialization.** (a) Hyperfine states (partially shown) of the ground and the optically excited manifold, and state transitions occurring during a single initialization cycle. Optical pulses are applied sequentially on the spin-flip transitions ($\Delta m_I = -1$, red arrows) and swap the population between the respective states with the probability p_i ($i \in \{1, ..., 7\}$). In between the pulses, the system can spontaneously relax from the excited to the ground state (dashed gray arrows), predominantly via a spin-preserving transition with branching probability β_i ($i \in \{0, ..., 7\}$). The green arrow indicates the optical transitions on which the population is probed after N_{init} initialization cycles. (b) Spin transfer probability to the $|-7/2\rangle_g$ state as a function of N_{init}, for different initial states (from left to right: $|-5/2\rangle_g$ to $|+7/2\rangle_g$). Solid lines are fits to the model explained in (a), and fit parameters p_i are shown in the inset.

B. Initialization in arbitrary nuclear spin states

Besides initialization in $|-7/2\rangle_g$, as explored above, any of the eight nuclear spin states can be initialized by combining spin-flip transitions from both the red and blue sidebands. This is demonstrated in Fig. S10 for four different hyperfine states.

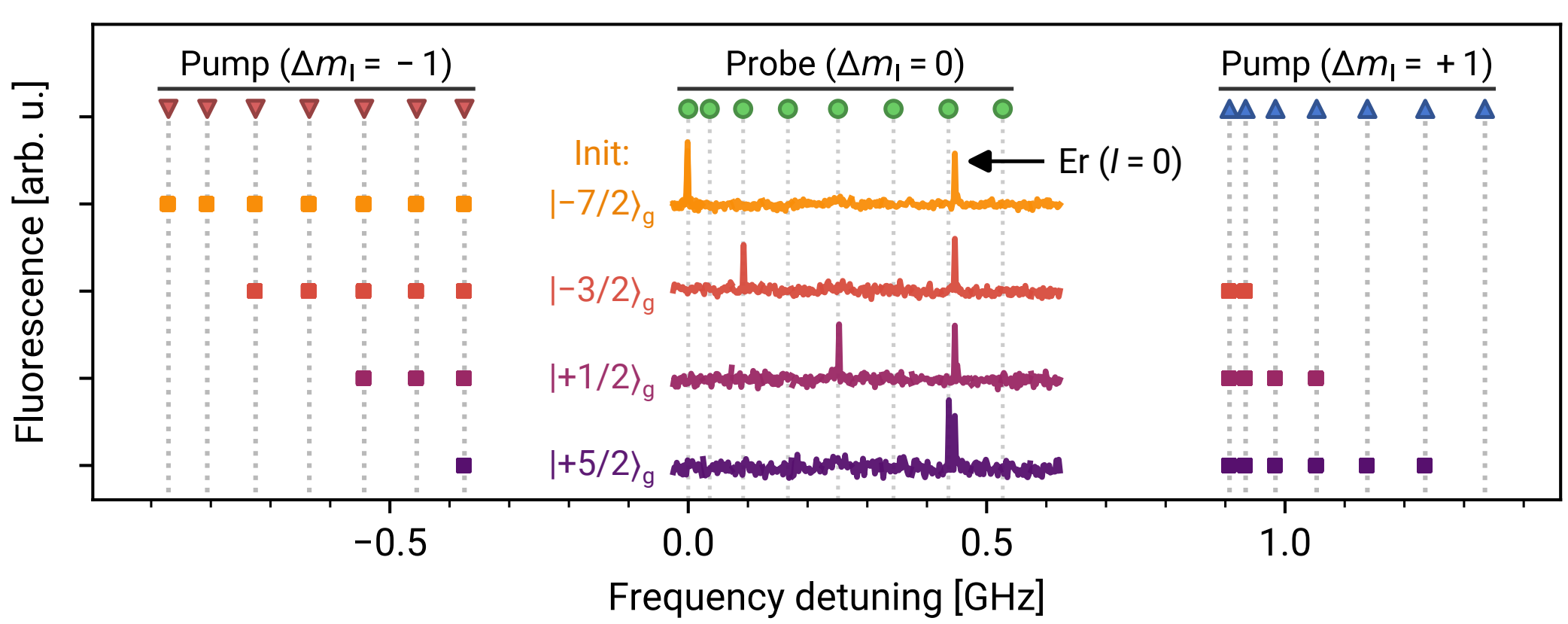

Supplementary Fig. S10. **Optical initialization in arbitrary nuclear spin states.** Fluorescence spectra showing a single ^{167}Er dopant in the outermost red-detuned satellite line ($\Delta^{|-7/2\rangle}_{|-7/2\rangle} = 195\,070.01$ GHz) that is initialized into different hyperfine ground states (indicated in the panel). We use optical spin pumping on the corresponding spin-flip transitions with $N_{\mathrm{init}} = 500$ cycles. Optical frequencies of the spin-preserving and spin-flip transitions are marked with circles and triangles, as described in Fig. S5. When optical pump pulses are applied to the spin-flip transitions marked by the colored squares, a narrow spectral feature appears on the associated spin-preserving transition. An Er dopant without hyperfine structure ($I = 0$) that is also present in the probed spectral window is used to normalize the detected fluorescence. All spectra are vertically offset for clarity.

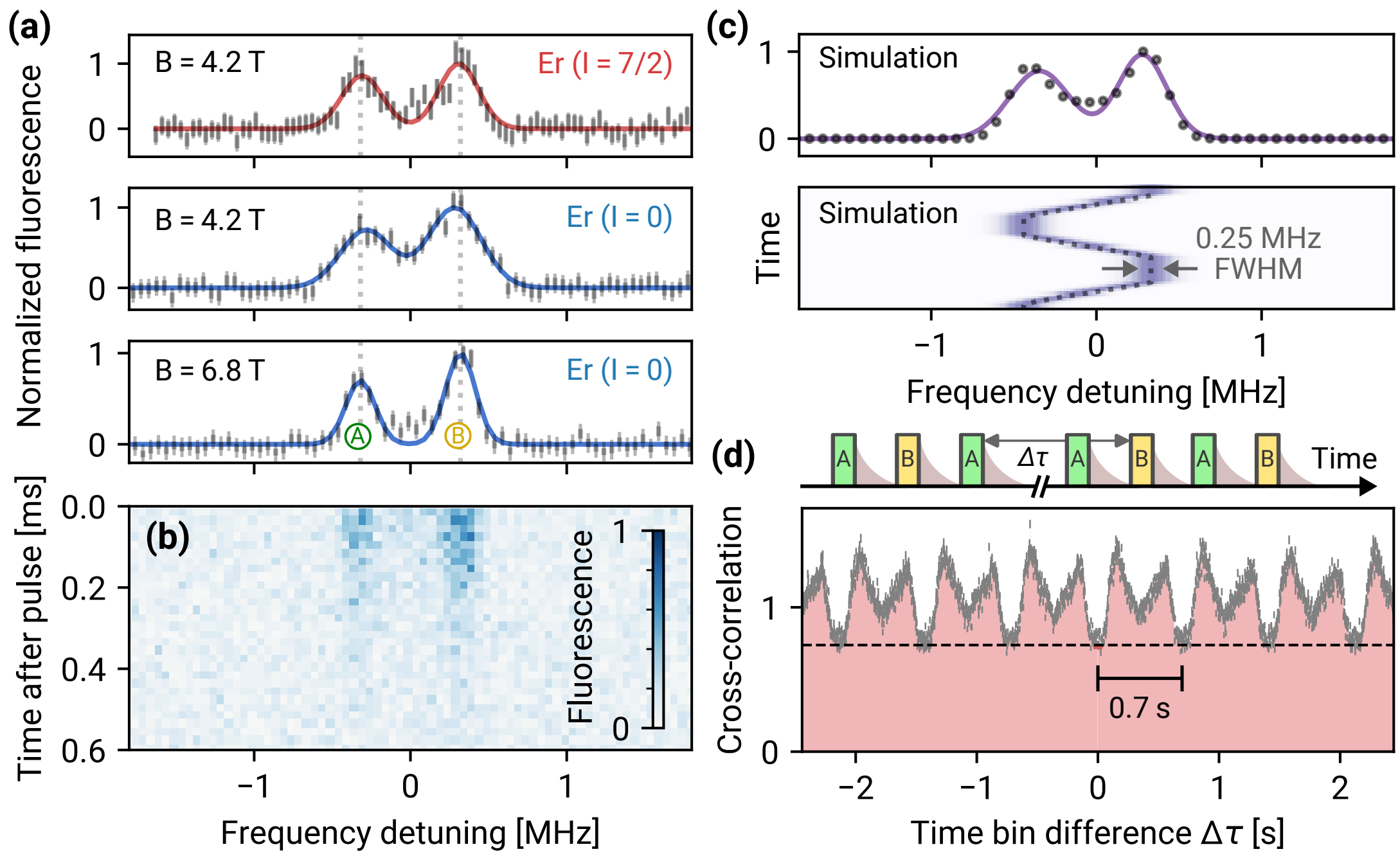

Supplementary Fig. S11. **Optical spectral diffusion at large magnetic fields.** (a) Fluorescence spectra of an ^{167}Er dopant initialized in the $|-7/2\rangle_g$ state (top panel) and two Er dopants with $I = 0$ (middle and bottom panel). A magnetic field of 4.2 or 6.8 T is applied along the b-axis of the YSO crystal. The spectral line shape of each dopant is then approximated by two Gaussian functions (solid line), where the linewidth is consistent with the expected broadening owing to the bath of Y nuclear spins, and the line splitting is attributed to magnetic field fluctuations caused by mechanical vibrations. (b) Time-resolved fluorescence spectrum of the dopant in the bottom panel of (a), recorded as a function of the time delay after the excitation pulse. Both spectral peaks ("A" and "B") exhibit similar optical lifetimes. (c) Assumed periodic modulation of the optical transition frequency (bottom panel, dotted line), and the resulting simulated time-averaged fluorescence intensity (top panel). Using a Gaussian linewidth of 0.25 MHz, the solid line in the top panel closely reproduces the measured spectral features in (a). (d) Cross-correlation of the fluorescence signals probed alternately on the two distinct spectral peaks ("A" and "B") in the bottom panel of (a). The observed oscillation period of 0.7 s coincides with the operation cycle of the pulse tube in the cryostat. The dashed black line indicates the detector dark count level in this measurement. Error bars: 1 SD.

IV. SPECTRAL DIFFUSION OF THE OPTICAL TRANSITIONS

The optical transitions of single Er dopants in YSO exhibit spectral diffusion with a Gaussian linewidth on the order of a few hundred kHz, resulting from the nuclear spin bath [2, 12]. However, in the current setup, an additional broadening is observed at large magnetic fields, which is attributed to fluctuations of the external magnetic field at the position of the dopants. These are characterized in the following via resonant optical excitation and fluorescence detection.

To this end, we focus on the spectral lineshape of three individual dopants shown in Fig. S11a, which are probed using narrowband optical pulses with a Gaussian envelope of 8 µs FWHM. All three dopants, including one ^{167}Er dopant in the $|-7/2\rangle_g$ state, exhibit a similar lineshape in both investigated magnetic field configurations, $B = 4.2$ T and 6.8 T. The lineshapes are characterized by two distinct peaks, which can be approximated by two Gaussian functions (solid lines). These peaks are attributed to the same emitter, as confirmed by independently measured, background-corrected autocorrelation functions $g^{(2)}(0) < 0.5$ when using broadband optical pulses addressing both peaks simultaneously. In addition, both peaks feature a similar optical lifetime, which is shown as a function of the frequency detuning in Fig. S11b. In all three cases, the frequency splitting of the two peaks is on the order of ≈ 0.5 MHz despite the differing magnetic field strengths. This can be explained by the reduced magnetic field sensitivity on the optical transition in this regime as a result of the mixing of adjacent crystal-field levels.

To gain insight into the dynamics of the spectral diffusion process, the two resolvable spectral peaks are probed alternately. The resulting cross-correlation function of the detected photons, shown in Fig. S11d, reveals that the optical transition frequency oscillates between the two values with a period of ≈ 0.7 s, which coincides with the pulse tube repetition period of the cryostat. Such periodic behavior is not expected for coupling to a spin-1/2 system, and is instead attributed to periodic fluctuations of the external magnetic field. Since the magnet was operated as a closed

superconducting loop ("persistent mode"), the generated magnetic field is not expected to show temporal variations. However, the field is not perfectly homogeneous, such that a spatial displacement would result in a variation of the magnetic field.

The measured spectral lineshape in Fig. S11a is qualitatively reproduced in panel (c), where we assume a clipped sinusoidal modulation of the transition frequency, in addition to a Gaussian broadening of 0.25 MHz arising from the bath of yttrium nuclear spins in YSO [13]. An asymmetry is introduced by assuming different clipping thresholds at lower and upper frequency detunings.

Assuming a purely magnetic contribution to the spectral broadening, the optical splitting can be used to estimate the magnitude of the field fluctuations at the position of the emitter. Here, the magnetic field sensitivity of the transition is derived from the crystal-field Hamiltonian [11], yielding a peak-to-peak field variation between 0.03 mT and 0.3 mT at a bias field of $B = 6.8\,\mathrm{T}$ along the b-axis. This corresponds to a relative field change of up to 4×10^{-5}, for which a perturbation along each of the axes (D1, D2, b) can be considered. While the spectral measurement does not provide the perturbation direction, we consider the most likely case a variation along the b-axis, which coincides with the axis of the bias field. A possible explanation for this motion is that elastomer-based dampers (NewPort M-ND01-A) contract and expand along the gravitational axis that is parallel to the magnetic field in response to the vibrations induced by the pulse tube. These vibrations are not damped out as their period of 0.7 Hz is below the eigenfrequency of the dampers, which is approximately 20 Hz. In Sec. VI C 3, we will use this result to estimate the variation of the hyperfine transition frequency, finding that it would significantly reduce the hyperfine coherence in agreement with our measurements.

On longer timescales, a slow drift of the optical transition frequency on the order of several MHz per week was observed. This shift is attributed to the damping of the current in the superconducting solenoid magnet. To compensate for this drift, the frequency of the $|{-7/2}\rangle_g \leftrightarrow |{-7/2}\rangle_e$ transition was periodically measured, and the spectroscopy laser was adjusted accordingly.

V. SINGLE-SHOT READOUT

A. Definition of the readout fidelity

The objective of the readout operation is to correctly identify the qubit state that is encoded in $|{-7/2}\rangle_g$ and $|{-5/2}\rangle_g$. To this end, we probe the transition $|{-7/2}\rangle_g \leftrightarrow |{-7/2}\rangle_e$. Fluorescence is only detected if the emitter is in state $|{-7/2}\rangle$, which is thus referred to as the "bright" state, while all other states are "dark". Ideally, the readout pulses are repeated until the number of photons detected in the bright state always exceeds a threshold that in turn is never surpassed when the qubit is in the dark state.

However, experimental imperfections may hinder a full separation of the distributions, limiting the fidelity of correctly identifying either of the two states. Thus, we define the readout fidelity as the minimum probability of obtaining the correct outcome, denoted by P_0 and P_1, for the states $|{-7/2}\rangle_g$ and $|{-5/2}\rangle_g$, respectively:

$$\mathcal{F} = \min\{P_0, P_1\}. \tag{5}$$

A photon threshold number that is too low or too high can increase the detection fidelity of one state, but will reduce the fidelity of the other one. Therefore, to maximize the fidelity, the threshold is chosen such that P_0 and P_1 are of similar magnitude.

B. Cyclicity of the transitions

In the experiment, achieving a high readout fidelity requires both efficient photon detection and a cyclic optical transition. The cyclicity refers to the average number of spontaneous decays from the excited state before the spin flips into a different state [14]. To quantify this, we define the average number of excitation attempts N_i on transition i, after which the fluorescence intensity, initially $I_i(0)$, has decreased to $I_i(N_i)/I_i(0) = 1/e$. After an excitation pulse, a spin flip can only occur if (a) the spin is in the excited state and (b) the emission occurs on the spin-flip transition before the next pulse, which would bring the emitter back to the ground state. The cyclicity can therefore be expressed in terms of the decay parameter N_i as follows:

$$\mathcal{C}_i = p_{\mathrm{em}} p_{\mathrm{exc}} N_i, \tag{6}$$

where p_{em} describes the emission probability and p_{exc} is the average probability to find the emitter in the excited state immediately after an excitation attempt.

Conversely, the cyclicity can also be related to the branching ratio $\beta_i = 1 - \beta_{\text{flip},i}$ of the spin-preserving transition using:

$$\mathcal{C}_i = -\frac{1}{\log(1-\beta_{\text{flip},i})}. \tag{7}$$

By definition, N_i is always greater than or equal to the intrinsic cyclicity $\mathcal{C}_i$. Correspondingly, we define the average spin-flip probability per excitation attempt as:

$$p_{\text{flip},i} = 1 - \exp(-1/N_i), \tag{8}$$

which effectively sets an upper limit to the number of excitation pulses that can be applied during a readout attempt before the fidelity starts to decrease.

In principle, the branching ratio can be derived from the crystal-field Hamiltonian. One expects that the value of $\mathcal{C}_0 \approx 8$ (using $\beta_{\text{flip},0} = 0.12$ from Fig. S4b) for an emitter in free space can be strongly enhanced by the Purcell effect. However, a quantitative prediction would require extrapolating the model in Ref. [11] to the field regime of the present experiment, which is not expected to provide the required accuracy. Thus, the cyclicity needs to be determined by measurements.

To this end, we focus on a single ^{167}Er dopant with an optical lifetime $T_1 = 0.296(7)$ ms. After spin initialization, we measure the fluorescence intensity as a function of the pulse number. The result is shown in Fig. S12a. We then determine the average number of excitation attempts after which the population in the prepared state decays to $1/e$, finding $N_0 = 269(4)$ in the $|-7/2\rangle$ state, and $N_1 = 124(2)$ in the $|-5/2\rangle$ state. In Fig. S12b, the fluorescence is integrated over the full optical lifetime after each of the first 1500 pulses. After normalization, the relative fluorescence intensity yields a measure of the transition cyclicity, which is largest for the spin-preserving transitions of the $|-7/2\rangle$ and $|+7/2\rangle$ states. The relative decrease on the other transitions is consistent with the prediction from the spin Hamiltonian. When comparing the obtained values to the cyclicity in free space, the measurement proves that the Fabry-Perot cavity strongly enhances the cyclicity of the spin-preserving transitions, as required for single-shot readout.

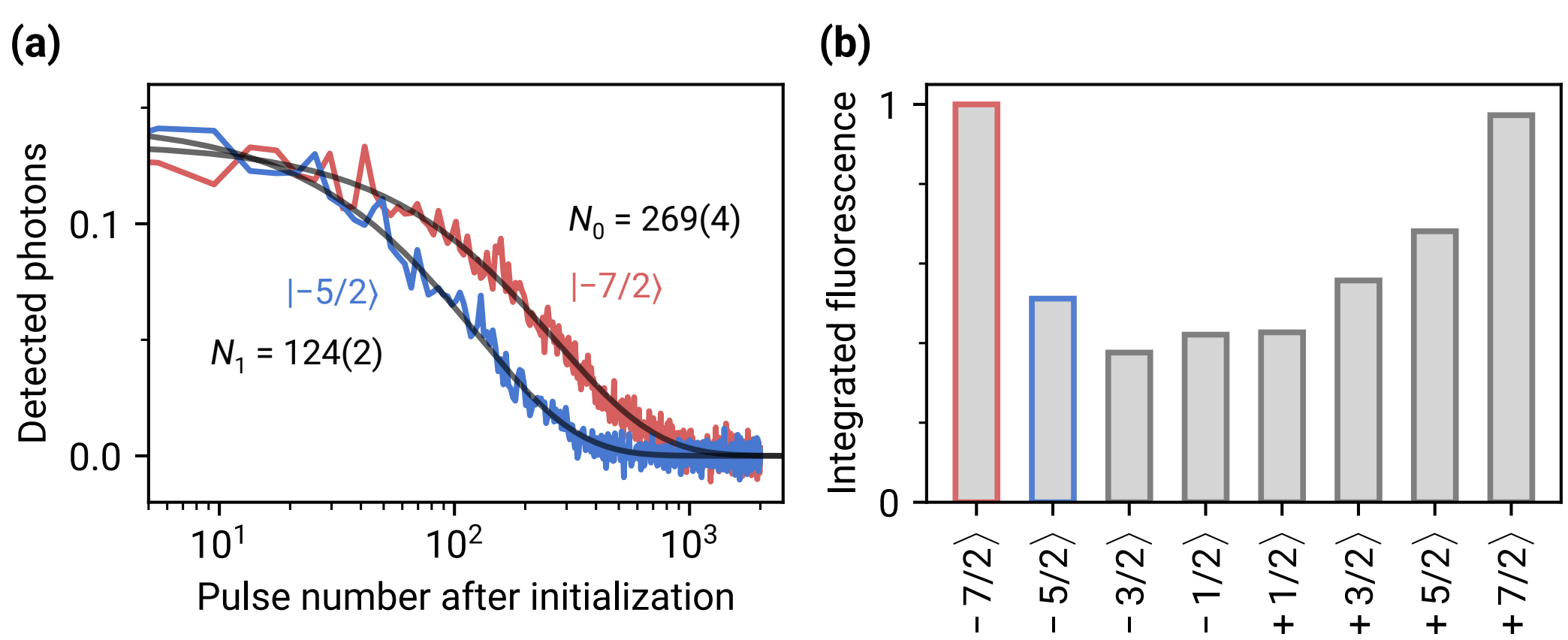

Supplementary Fig. S12. **Cavity-enhanced cyclicity of the optical transitions.** (a) Fluorescence detected on the spin-preserving transitions of an ^{167}Er dopant, optically initialized in the $|-7/2\rangle$ (red) or $|-5/2\rangle$ (blue) state, as a function of the number of excitation pulses. The probed transitions exhibit an effective Purcell enhancement of $P = 38.6(9)$ and 35(3), respectively, when on resonance with the cavity. The black solid lines indicate a fit to an exponential function, yielding the average number of excitation attempts N_0 and N_1 after which the initial population decays to $1/e$. (b) Integrated fluorescence in a time interval of 1.5 ms after the first 1500 pulses, on each spin-preserving optical transition. The fluorescence intensity, normalized to the strongest transition, $|-7/2\rangle_g \leftrightarrow |-7/2\rangle_e$, represents the relative cyclicity of the individual transitions. In both panels, a magnetic field of $B = 6.8$ T is applied along the b-axis of the YSO crystal.

C. Limitation of the readout fidelity

For an average spin-flip probability $p_{\rm flip} := p_{\rm flip,0}$, the relative fluorescence intensity after the nth excitation pulse is given by:

$$I(n) = p_{\rm init} p_{\rm exc} p_{\rm em} p_{\rm det} (1 - p_{\rm flip})^n, \tag{9}$$

where $p_{\rm init}$ is the probability to find the spin initially in the bright state, and $p_{\rm det}$ denotes the single-photon detection efficiency.

We assume that the entire readout sequence is much shorter than the spin relaxation time, such that only optical pumping contributes to spin flips. The probability distribution of recording in total $N_{\rm C}$ counts after $N_{\rm P}$ excitation attempts will then follow a Poisson binomial distribution. Additionally, the experimentally measured distributions also contain a contribution from detector dark counts.

In the absence of detector dark counts, the spin readout fidelity is limited by the finite detection efficiency and imperfect cyclicity of the optical transition. If the emitter is in the bright state but no photons are detected in $N_{\rm P}$ excitation attempts, the spin state will be inferred erroneously. This sets an upper bound on the achievable fidelity:

$$\mathcal{F}_{\rm max} = 1 - [(1 - p_{\rm det})(1 - p_{\rm flip})]^{N_{\rm P}} - \sum_{n=0}^{N_{\rm P}-1} p_{\rm flip} (1 - p_{\rm det})^n (1 - p_{\rm flip})^n \tag{10}$$

$$= 1 - \frac{p_{\rm det}(1 - p_{\rm det})^{N_{\rm P}} (1 - p_{\rm flip})^{N_{\rm P}+1} + p_{\rm flip}}{p_{\rm det}(1 - p_{\rm flip}) + p_{\rm flip}}. \tag{11}$$

This equation includes the cases when the spin remains in the bright state throughout the readout but no photons are detected, and when a spin flip occurs after the $(n + 1)$th excitation attempt, given that no photons were detected in the preceding attempts. The corresponding readout error, $1 - \mathcal{F}_{\rm max}$, is shown in Fig. S13a as a function of $N_{\rm P}$, using the highest detection efficiency achieved in this work ($p_{\rm det} = 0.16$). The saturation value for $N_{\rm P} \to \infty$ and the same detection efficiency is shown in Fig. S13b.

If dark counts occur during readout, the maximum fidelity given by Eq. 11 may not be achievable. For an average number of dark counts $\bar{n}_{\rm dc}$ accumulated during the entire readout, the probability of recording k dark counts is described by a Poisson distribution:

$$P_{\rm dc}(k) = \frac{\bar{n}_{\rm dc}^k}{k!} \exp(-\bar{n}_{\rm dc}). \tag{12}$$

As a consequence, the overlap of the photon-counting histograms for the bright and dark states is increased, which reduces the achievable readout fidelity further.

Based on this analysis, assuming detector dark counts can be reduced sufficiently, one can derive an upper limit for the fluorescence-based single-shot readout fidelity in our experiment, depending on the number of excitation pulses and the remaining probability of a spin flip, as shown in Fig. S13. Clearly, minimizing the error probability requires a small flip probability. This is achieved in our experiment by a strong and selective Purcell enhancement of the readout transition. For a flip probability $p_{\rm flip} = 0.00274(3)$ on the $|{-7/2}\rangle_e \to |{-7/2}\rangle_g$ transition, the fidelity limit as given by Eq. 11 is $\mathcal{F}_{\rm max} = 0.983(1)$.

D. Spin initialization fidelity

Additionally, we estimate the reduction in the effectively measured readout fidelity, resulting from incorrect spin initialization prior to each readout attempt. To this end, we analyze the intensity autocorrelation function of the ^{167}Er emitter after pulsed resonant excitation on the corresponding spin-preserving optical transition, defined as:

$$g^{(2)}(\Delta n) = \frac{\langle I(n) I(n + \Delta n)\rangle_n}{\langle I(n)\rangle_n^2}, \tag{13}$$

where Δn denotes the distance between two time bins. The detected intensity in the nth bin when the spin is reinitialized after every $(bN_{\rm P})$th pulse with $b \in \mathbb{N}$ denoting the readout block index reads:

$$I(n) = p_{\rm init} p_{\rm exc} p_{\rm em} p_{\rm det} (1 - p_{\rm flip})^{n \bmod N_{\rm P}}. \tag{14}$$

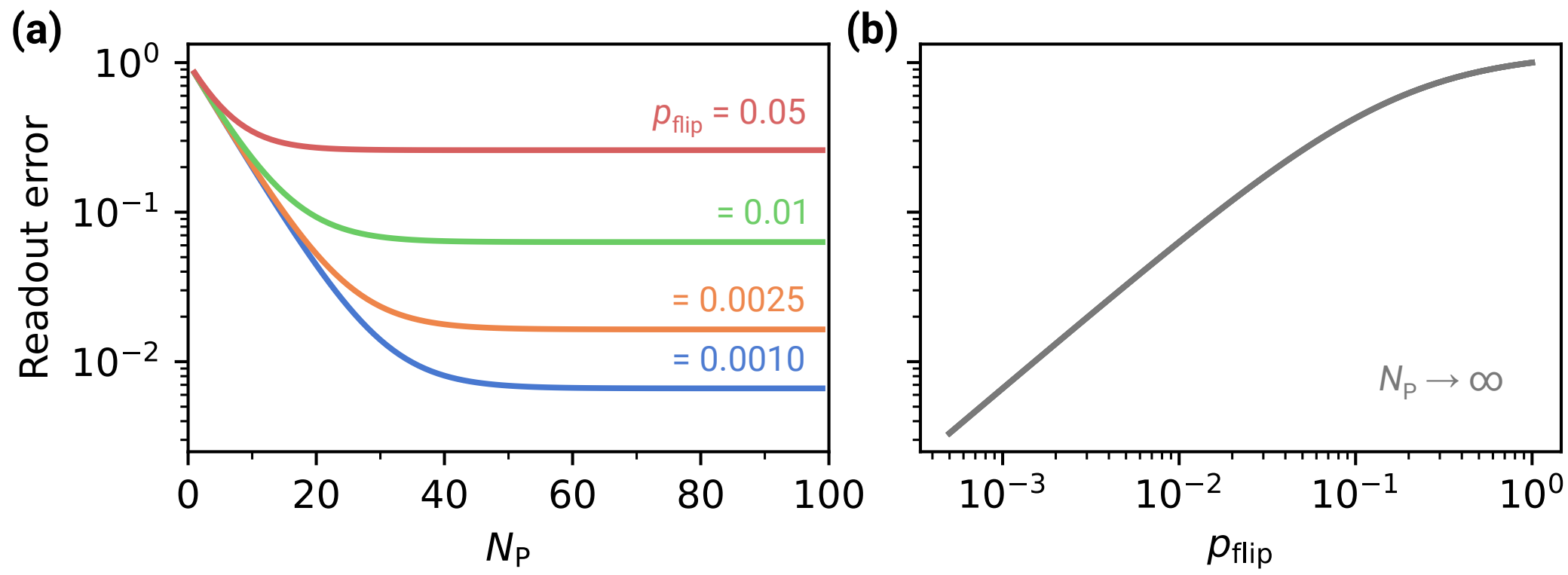

Supplementary Fig. S13. **Fidelity limit of a fluorescence-based readout.** (a) Minimum achievable readout error, $1-\mathcal{F}_{\max}$ (see Eq. 11), as a function of the number of excitation pulses N_P for different spin-flip probabilities p_{flip}. (b) Readout error as a function of p_{flip} for $N_\text{P} \to \infty$. In both panels, the detection efficiency is fixed at $p_{\text{det}} = 0.16$, and the detector is assumed to be ideal, not contributing any dark counts.

Here, 'mod' refers to the modulo division. After evaluating Eq. 13 under the assumption $p_{\text{em}} \approx 1$, we arrive at:

$$g^{(2)}(\Delta n) = \left[\frac{p_{\text{flip}}}{1-(1-p_{\text{flip}})^{N_\text{P}}}\right]^2 \frac{N_\text{P}^2}{N-\Delta n} \sum_{n=0}^{N-\Delta n-1} (1-p_{\text{flip}})^{(n+\Delta n) \bmod N_\text{P}} \times \begin{cases} 0, & \text{if } \Delta n = 0 \\ (p_{\text{init}})^{-1}, & \text{if } n \bmod N_\text{P} + \Delta n < N_\text{P} \\ (1-p_{\text{flip}})^{n \bmod N_\text{P}}, & \text{otherwise} \end{cases} \tag{15}$$

Notably, this expression has no explicit dependence on p_{exc} and p_{det} and allows us to infer p_{init}. In the presence of detector dark counts, the autocorrelation function is rescaled by an offset Θ, which can be treated as a free parameter or obtained from the signal-to-noise ratio as outlined in Ref. [15]. Thus, the autocorrelation function obtained in the experiment is given by:

$$\tilde{g}^{(2)}(\Delta n) = g^{(2)}(\Delta n) \cdot (1-\Theta) + \Theta. \tag{16}$$

The predicted autocorrelation function is shown in Fig. S14a for different initialization fidelities p_{init}. For imperfect spin initialization, $p_{\text{init}} < 1$, the emitted photons will exhibit an increased bunching compared to ideal spin initialization ($p_{\text{init}} = 1$, solid blue line), if both photons are detected in the same readout block ($\Delta n < N_\text{P}$). Photons detected in separate readout blocks ($\Delta n \geq N_\text{P}$), however, are not correlated with each other, as the spin initialization is assumed to be fully probabilistic. For N_P larger than the average spin population decay constant N_0, additional bunching features appear at $\Delta n = bN_\text{P}$ (vertical dotted lines), which are independent of p_{init}.

We next consider the experimental dataset shown in the photon counting histogram in Fig. 3a for the bright state ($|-7/2\rangle_g$, orange bars). Evaluating the corresponding $\tilde{g}^{(2)}(\Delta n)$ without background correction, as shown in Fig. S14b (gray bars), we find that the data nearly matches the theoretical prediction (Eq. 16) for $p_{\text{init}} = 1$. Here, we also use the previously determined average spin-flip probability $p_{\text{flip}} = 0.00274(3)$, and an offset $\Theta = 0.47$, which is obtained from independently measured detector dark counts. To retrieve the spin initialization fidelity from the measured autocorrelation function, we perform a fit to Eq. 16, while keeping p_{init} as a free parameter. This yields a bright-state initialization fidelity of $p_{\text{init}} = 0.973(9)$. Thus, we conclude that the initialization error contributes approximately 30 % to the uncorrected readout fidelity $\mathcal{F} = 91(2)$ %.

VI. COHERENT CONTROL WITH RAMAN PULSES

In the following, we explain the techniques used for coherent control of the ground state in more detail. Due to the small magnetic dipole moment of the nuclear spin, resonant microwave/RF driving would require high power levels, which can potentially cause undesired heating of the experimental setup. This, in turn, may reduce the coherence or detune the resonance of the optical cavity due to thermal expansion. Thus, instead we implement coherent control via optical Raman fields whose frequency difference of ≈ 0.9 GHz matches the ground-state level splitting and lies in a frequency range that can be readily reached with commercially available electro-optical modulators.

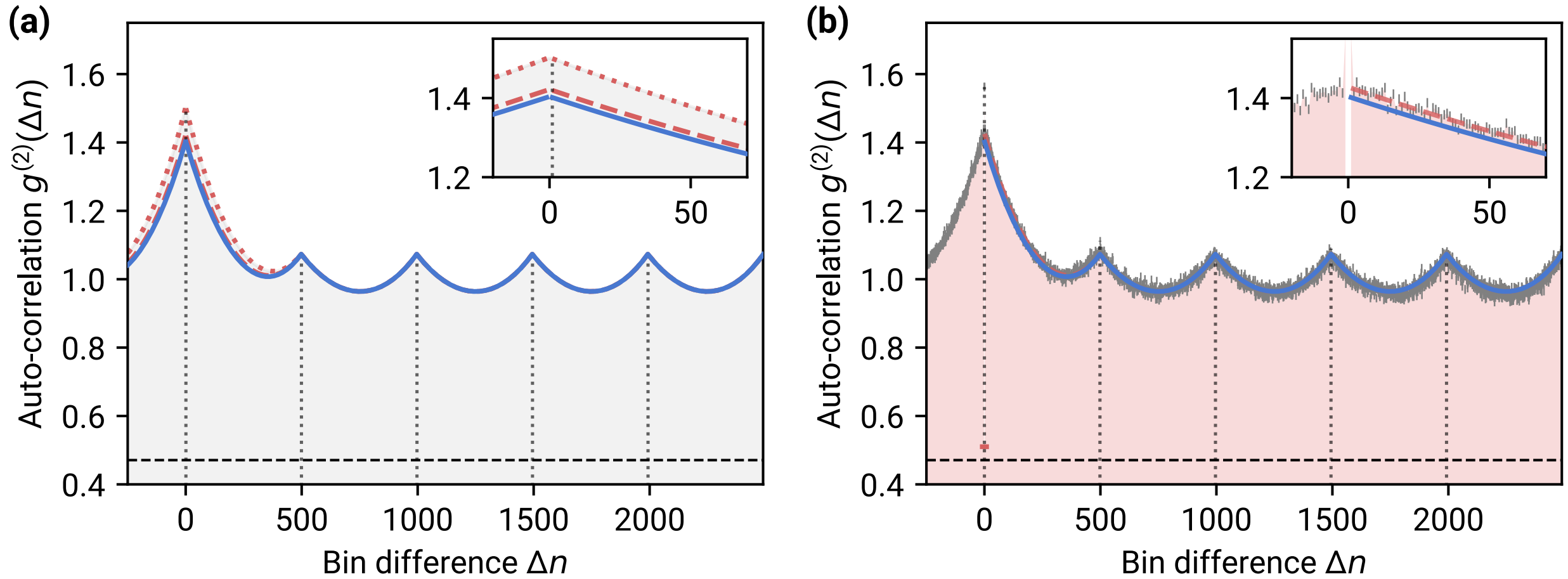

Supplementary Fig. S14. **Nuclear-spin initialization fidelity.** (a) Calculated intensity autocorrelation function of a periodically initialized emitter ($N_\mathrm{P} = 500$, vertical dotted lines). The detection of a photon heralds the bright state, thereby increasing the probability of detecting a second photon within the same readout block. This results in a bunching feature for $\Delta n < N_\mathrm{P}$ ($p_\mathrm{init} = 1$, blue dashed line), which becomes more pronounced for imperfect spin initialization: $p_\mathrm{init} = 0.98$ (red dashed line) and $p_\mathrm{init} = 0.9$ (dotted red line). The horizontal dashed line indicates the background due to detector dark counts. (b) Measured intensity autocorrelation function (gray bars) on the $|-7/2\rangle_g \leftrightarrow |-7/2\rangle_e$ transition with initialization into the $|-7/2\rangle_g$ state. The expected autocorrelation function for perfect initialization ($p_\mathrm{init} = 1$) is shown as a solid blue line. The slightly increased bunching in the measured data for $\Delta n < N_\mathrm{P}$, is fit to Eq. 16 (red dashed line), yielding a bright state initialization fidelity of $p_\mathrm{init} = 0.973(9)$. The sharp bunching feature at $\Delta n = 1$ is attributed to the increased optical excitation probability when a photon was detected in the previous bin, indicating that the emitter returned to the ground state. Due to an emission probability of $p_\mathrm{em} = 0.82(1)$, a photon is not emitted on every excitation attempt, and the emitter may be optically de-excited by the subsequent pulse, effectively reducing the average excitation probability. Thus, the bins $\Delta n \leq 2$ are excluded from the fit. Error bars: 1 SD.

A. Stimulated Raman transitions: Theory

In the following, we approximate the hyperfine structure of an ^{167}Er dopant as a three-level system and assume that it is driven by classical fields. To induce a stimulated Raman transition, two control fields E_1 and E_2 with the complex Rabi frequencies Ω_1 and Ω_2 off-resonantly couple the ground states $|g_1\rangle$ and $|g_2\rangle$ to the common intermediate state $|e_1\rangle$, forming a Λ-type configuration.

Under the rotating wave approximation and in the rotating frame, the interaction Hamiltonian in the basis $\{|g_1\rangle, |e_1\rangle, |g_2\rangle\}$ takes the form:

$$\mathcal{H}_\mathrm{R} = h \begin{pmatrix} 0 & \Omega_1^*/2 & 0 \\ \Omega_1/2 & \Delta & \Omega_2^*/2 \\ 0 & \Omega_2/2 & \delta_\mathrm{R} \end{pmatrix}. \tag{17}$$

Here, Δ and δ_R are the one-photon and two-photon detunings, respectively. All quantities are expressed in frequency units (Hz). Spontaneous emission is not included in the Hamiltonian.

In the presence of the control fields, the energy levels experience a shift due to the AC Stark and/or Zeeman effect. These effects may appear simultaneously, as the transitions under investigation exhibit mixed electric and magnetic dipole moments. Their magnitude depends on the Rabi frequency of the respective transition and the one-photon detuning, and is given by:

$$\delta_\mathrm{AC1} = \frac{|\Omega_1|^2}{4\Delta}, \quad \delta_\mathrm{AC2} = \frac{|\Omega_2|^2}{4\Delta}, \tag{18}$$

assuming that cross-couplings, i.e., shifts induced by E_1 (E_2) on the level $|g_2\rangle$ ($|g_1\rangle$), are negligible.

For $\Omega_1 \neq \Omega_2$, the resulting differential shift alters the two-photon resonance condition ($\delta_\mathrm{R} = 0$), which can be expressed as:

$$\nu_2 - \nu_1 = \delta + (\delta_\mathrm{AC2} - \delta_\mathrm{AC1}). \tag{19}$$

Here, $\nu_{1,2}$ are the carrier frequencies of the control fields $E_{1,2}$ and δ denotes the unperturbed ground-state splitting. A frequency shift due to momentum transfer when a photon is absorbed or emitted can typically be neglected, as the transferred momentum is absorbed by the entire lattice. However, the differential AC Stark and Zeeman shifts must be carefully stabilized to prevent uncontrolled dephasing.

If the one-photon detuning Δ from the intermediate state is large compared to the Rabi frequencies, $|\Delta| \gg \Omega_{1,2}$, the population of the intermediate state remains negligible during the pulse. Under these conditions, the intermediate state can be adiabatically eliminated [16], allowing the system to be treated as a two-level system with an effective Rabi frequency given by:

$$\Omega_\mathrm{R} = \frac{\Omega_1^* \Omega_2}{2\Delta}. \tag{20}$$

Here, Ω_1 and Ω_2 are complex Rabi frequencies, and their phase difference determines the effective phase of the Raman pulse.

When a square-shaped Raman pulse is applied, i.e., when the Rabi frequencies Ω_1 and Ω_2 are constant in time, the effective two-level system undergoes Rabi oscillations. For a system initially prepared in the lower ground-state $|g_1\rangle$, the population of the upper ground-state $|g_2\rangle$ after a pulse of duration τ_p is given by:

$$P_{|g_2\rangle}(\tau_p) = \frac{\Omega_\mathrm{R}^2}{\widetilde{\Omega}_\mathrm{R}^2} \sin^2\left(\pi \widetilde{\Omega}_\mathrm{R} \tau_p\right), \tag{21}$$

with the generalized Raman Rabi frequency defined as $\widetilde{\Omega}_\mathrm{R} := \sqrt{|\Omega_\mathrm{R}|^2 + (\delta_\mathrm{R})^2}$. Since the two-photon resonance condition depends on both Ω_1 and Ω_2, the Raman pulse area will be chosen by varying the pulse length τ_p, while keeping the effective Rabi frequency constant.

B. Effect of the optical cavity on stimulated Raman transitions

As the used experimental setup does not allow side access, optical fields can only be coupled to the emitter via the cavity mode. In the following, we treat the optical fields classically and assume that the cavity field reaches its steady state on timescales much shorter than the emitter-photon interaction. In the presence of an optical cavity, stimulated Raman transitions are affected in two ways: First, one or both control fields may be detuned from the cavity resonance and therefore experience attenuation. Second, fluctuations in the cavity resonance frequency (see Sec. I D) can modulate the amplitude and phase of the control fields at the emitter. This leads to unequal AC Stark and Zeeman shifts of the two ground states, resulting in a random detuning of the two-photon resonance. To quantify these effects, we analyze the dependence of the effective Raman Rabi frequency Ω_R and its sensitivity on the cavity detuning Δ_C, where Δ_C is defined relative to the spin-preserving transition of the emitter (see Fig. S15a).

The Rabi frequencies on resonance with the cavity mode for the two optical transitions are defined as $\Omega_1^{(0)}$ and $\Omega_2^{(0)}$, respectively. When detuned from the cavity resonance, these Rabi frequencies are reduced according to:

$$\Omega_{1,2} = \Omega_{1,2}^{(0)} \frac{i\kappa/2\pi}{i\kappa/2\pi - \Delta_\mathrm{C}^{(1,2)}}. \tag{22}$$

Here, κ is the cavity field decay rate, and $\Delta_\mathrm{C}^{(1,2)}$ is the detuning of each control field to the cavity resonance. Using the experimental parameters, $\kappa = 2\pi \times 33\,\mathrm{MHz}$, $\Delta = -0.1\,\mathrm{GHz}$ and a ground-state splitting $\delta = 0.9\,\mathrm{GHz}$, the relative effective Rabi frequency, $|\Omega_\mathrm{R}/\Omega_\mathrm{R}^{(0)}|$ with $\Omega_\mathrm{R}^{(0)} := \Omega_2^{(0)}\Omega_1^{(0)*}/(2\Delta)$, is plotted in Fig. S15b. This function is symmetric around the center frequency of the two control fields, $(\nu_1 + \nu_2)/2$, and does not depend on the ratio $\rho_\Omega := \Omega_1^{(0)}/\Omega_2^{(0)}$. On resonance with either of the control fields, the relative Rabi frequency can reach up to $\Omega_\mathrm{R}/\Omega_\mathrm{R}^{(0)} \approx 0.04$. However, if the cavity resonance fluctuates, it may be advantageous to choose a detuning that results in a lower effective Rabi frequency, as shown below.

We quantify the sensitivity of the effective Raman Rabi frequency to fluctuations in the cavity resonance, Δ_C, by the following derivative:

$$S_\mathrm{R} := \frac{1}{|\Omega_\mathrm{R}|} \left| \frac{\mathrm{d}(\widetilde{\Omega}_\mathrm{R})}{\mathrm{d}(\Delta_\mathrm{C})} \right|, \tag{23}$$

which is normalized to the effective Rabi frequency for a given cavity detuning Δ_C. Using the same parameters as above, the resulting sensitivity is plotted for different ratios $\rho_\Omega = 1, 10$ in Fig. S15c.

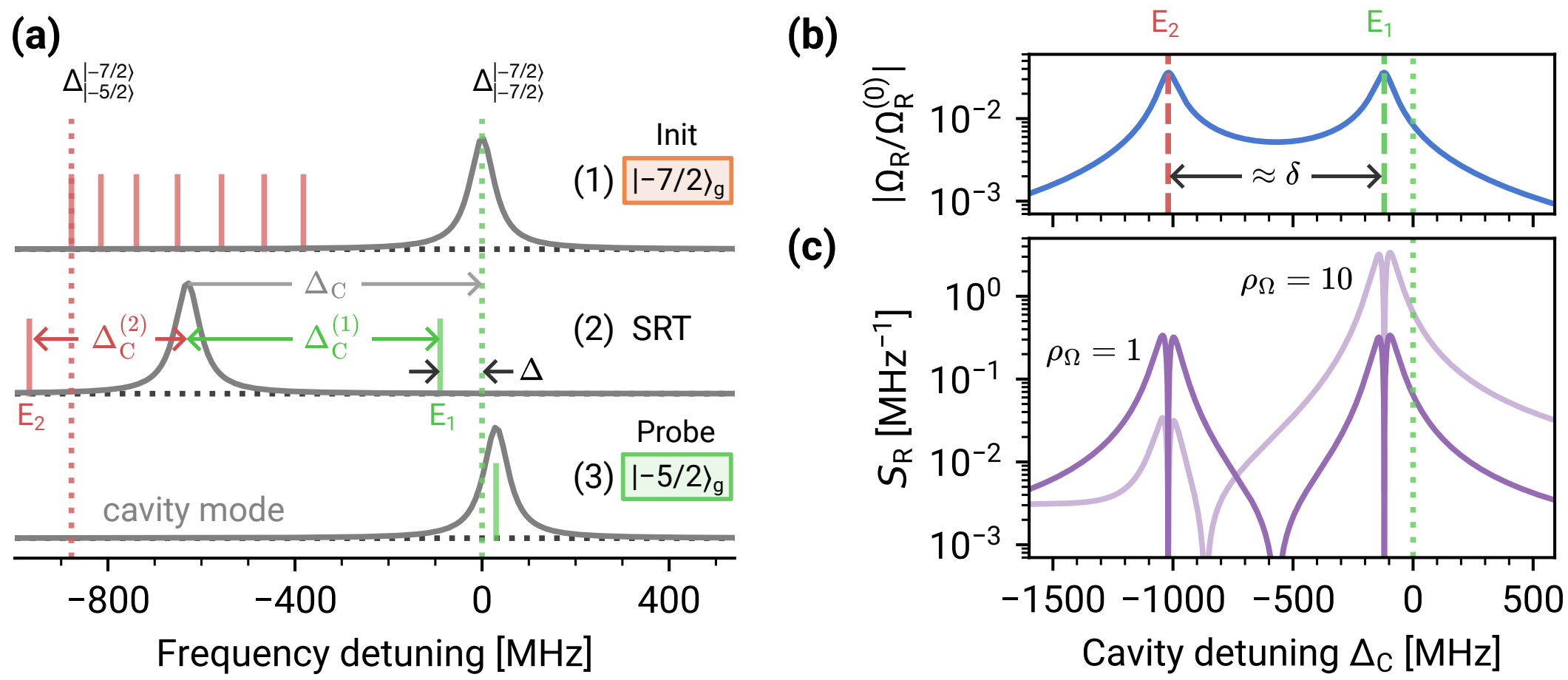

Supplementary Fig. S15. **Stimulated Raman transitions in an optical cavity.** (a) Visualization of the detunings used in the experimental sequence, including the cavity resonance (solid gray line, 65 MHz FWHM) and the frequencies of the applied optical fields (red and green vertical lines). The system is first initialized into the $|-7/2\rangle_g$ state (1, top) via optical pumping of the $\Delta m_I = -1$ spin-flip transitions (red lines), while the cavity is resonant with the lowest nuclear-spin-preserving transition (green dotted line). To induce a stimulated Raman transition (2, center), the cavity is detuned by Δ_C and two optical fields, E_1 and E_2, are applied with one-photon detuning Δ from the spin-preserving and spin-flip transitions, respectively. A negative cavity detuning, $\Delta_\mathrm{C} < 0$, corresponds to a red shift of the cavity resonance with respect to the spin-preserving transition. For optical readout (3, bottom), the cavity mode is tuned back into resonance with the spin-preserving transition. (b) Relative reduction of the Raman Rabi frequency in the effective two-level system ($|-7/2\rangle_g \leftrightarrow |-5/2\rangle_g$) as a function of the cavity detuning Δ_C. The normalization factor $\Omega_\mathrm{R}^{(0)} := \Omega_2^{(0)}\Omega_1^{(0)*}/(2\Delta)$ corresponds to a hypothetical situation in which both optical fields would be resonant with the cavity (which would require $\kappa/\pi \gg \delta$). (c) Relative sensitivity of the Rabi frequency to the cavity detuning, as defined in Eq. 23. To minimize pulse errors, one would operate at the minimum sensitivity. The optimal resonator detuning to achieve this depends on the ratio of the Rabi frequencies $\rho_\Omega := \Omega_1^{(0)}/\Omega_2^{(0)}$.

Low sensitivity values can be obtained at very large detuning; however, this would require impractical values of the laser power to maintain a sufficient Ω_R. Thus, we instead restrict the cavity resonance to the frequency range between ν_1 and ν_2, which corresponds to the condition $\Delta_\mathrm{C}^{(1)} + \Delta_\mathrm{C}^{(2)} < \delta$. For equal Rabi frequencies, $\rho_\Omega = 1$, the minimal sensitivity is achieved when the cavity resonance is tuned exactly to the center between the two control fields. When the Rabi frequencies differ, $\rho_\Omega \neq 1$, the optimal detuning shifts towards the frequency of the weaker control field.

In addition to modulating the amplitude of the driving fields, the cavity also affects their phase. Analogously, using the argument function arg(...), we define the phase sensitivity of the complex Rabi frequency to fluctuations in the cavity detuning as:

$$S_\mathrm{R}^{(\mathrm{phase})} := \left| \frac{\mathrm{d}(\arg(\Omega_\mathrm{R}))}{\mathrm{d}(\Delta_\mathrm{C})} \right| . \tag{24}$$

In contrast to the amplitude sensitivity, the highest phase sensitivity is found when either of the control fields is exactly resonant with the cavity mode (not shown). For sufficiently large detunings ($\Delta_\mathrm{C}^{(1,2)} \gg 0$), the behavior of $S_\mathrm{R}^{(\mathrm{phase})}$ resembles the amplitude sensitivity S_R for $\rho_\Omega = 1$. However, unlike the amplitude sensitivity, $S_\mathrm{R}^{(\mathrm{phase})}$ is independent of the Rabi frequency ratio ρ_Ω.

In conclusion, to minimize pulse errors when the cavity resonance is subject to fluctuations, one will choose similar one-photon Rabi frequencies, i.e., $\rho_\Omega \approx 1$. In this case, both the amplitude and phase sensitivity are minimized when the cavity mode is equally detuned from both control fields, $\Delta_\mathrm{C}^{(1)} \approx \Delta_\mathrm{C}^{(2)}$. This condition has been used in the experiments described in the main text.

C. Coherent population transfer between adjacent ^{167}Er hyperfine states

At large magnetic fields, a suitable Λ-system in an ^{167}Er dopant can be formed using one spin-preserving optical transition and one spin-flip transition with $\Delta m_I = \pm 1$. The latter exhibits a non-zero dipole moment, whereas higher-order spin-flip transitions are significantly weaker. Therefore, efficient population transfer using two control

fields is only feasible between adjacent hyperfine levels. In the following, we will focus on the qubit manifold and induce transitions between $|-7/2\rangle_g \leftrightarrow |-5/2\rangle_g$ via the excited state level $|-7/2\rangle_e$. To increase the separation from other hyperfine levels outside the Λ-system, the control field acting on the spin-preserving transition is red-detuned.

1. *Experimental sequence and Rabi oscillations*

Since the phase of a Raman pulse in the effective two-level system is determined by the phase difference between the two control fields (Eq. 20), it is essential that the relative phase remains stable throughout the experiment. To achieve this, a single-mode laser is modulated by an IQ-EOM to generate two single-sideband tones with a frequency splitting that matches the two-photon resonance. As both control fields originate from the same laser source and propagate along the same optical path, they will not experience a relative phase shift even in case of changes in the optical path that may, e.g., be induced by thermal fluctuations or vibrations.

As discussed in Sec. VI B, in an optical cavity whose resonance frequency is not perfectly stable, the highest Raman pulse fidelity is achieved when the cavity is detuned relative to both control fields. To still enable an optical readout through resonant fluorescence detection and efficient spin initialization, we employed the sequence depicted in Fig. S15a. First, the cavity mode (solid gray line) is tuned on resonance with a spin-preserving transition (dotted vertical line), and the nuclear spin is initialized by optical pumping on the red-detuned spin-flip transitions (red vertical bars). In the second step, before applying the Raman pulse, the cavity resonance is detuned by $\Delta_\mathrm{C} \approx -\delta/2$ by adjusting the voltage at the piezo-electric actuator and thereby changing the resonator length. This tuning process takes around 10 ms, being well within the hyperfine lifetime. Finally, the cavity resonance is tuned back to the spin-preserving transitions, and the spin population is probed on the corresponding transition (green vertical bar).

For driving stimulated Raman transitions, the detuning Δ should meet several conditions: First, it has to exceed the optical spectral diffusion linewidth: $|\Delta| \gg \Delta_\mathrm{SD} \approx 1\,\mathrm{MHz}$ to avoid fluctuations of the Rabi frequency. Second, to minimize the population of the intermediate state during the pulse, the condition $|\Delta| \gg |\Omega_\mathrm{R}|$ should be met. Finally, increasing Δ while keeping the effective Rabi frequency constant reduces the magnitude of AC Stark and Zeeman shifts. However, maintaining a high Rabi frequency at larger detunings also requires higher optical power, which can lead to heating of the crystal. To avoid this and to keep the control pulses short, a practical upper limit on Δ is imposed.

In our experiment, the splitting of the hyperfine levels has been determined by pump-probe measurements on individual dopants. However, the yet unknown AC Stark and Zeeman shifts will change the two-photon resonance condition. Therefore, the latter is experimentally determined by keeping ν_1 constant and sweeping the two-photon detuning δ_R via ν_2. Fig. S16a shows the population of the upper ground-state $|-5/2\rangle_g$ after applying a Raman pulse with varying δ_R and pulse length τ_p. With the effective Rabi frequency $\Omega_\mathrm{R} = 1\,\mathrm{MHz}$ as a free parameter, the experimental data is in good agreement with Eq. 21, plotted in Fig. S16b. The observed damping, which is not included in this model, will be discussed below using experimental datasets recorded under the two-photon resonance condition ($\delta_\mathrm{R} = 0$).

2. *Raman pulse fidelity and hyperfine dephasing time*

Experimental imperfections, such as laser pulse area fluctuations or dephasing between the laser and the emitter, can lead to a reduced fidelity of the qubit rotations. To quantify this, we use the data obtained in the Rabi oscillation measurement and perform a least-squares fit to a stretched-exponential decay with stretching factor n, $P_\mathrm{Rabi}(\tau_p) = \frac{1}{2}\{\cos(2\pi\Omega_\mathrm{R}\tau_p)\exp[-(\tau_p/T_d)^n]+1\}$. We then define the pulse fidelity as the relative decrease of the initial population after one oscillation period, $\tau_p = 1/\Omega_\mathrm{R}$, given by:

$$\mathcal{F}_\mathrm{R} = \frac{P_\mathrm{Rabi}(0) - P_\mathrm{Rabi}(1/\Omega_\mathrm{R})}{P_\mathrm{Rabi}(0)}. \tag{25}$$

To investigate the origin of the imperfections, we first investigate the pulse fidelity for different cavity detunings Δ_C and Rabi frequencies Ω_R, as shown in Fig. S16c. When the cavity detuning is varied while keeping Ω_R at a similar magnitude, we observe better fidelity for non-zero cavity detunings. This is expected from the model in Fig. S15c, because of residual fluctuations of the cavity resonance caused by cryostat vibrations. Thus, the highest fidelity among the investigated detunings is achieved for $\Delta_\mathrm{C} = -400\,\mathrm{MHz}$, yielding $\mathcal{F}_\mathrm{R} = 0.98$. The envelope in this case exhibits a non-exponential decay with a stretching factor $n = 1.8(6)$.

After determining the optimal cavity detuning, we proceed by varying the Raman Rabi frequency. The resulting Rabi oscillations, shown in Fig. S17a for an increasing Rabi frequency from (1) to (3), are again fitted to an oscillation

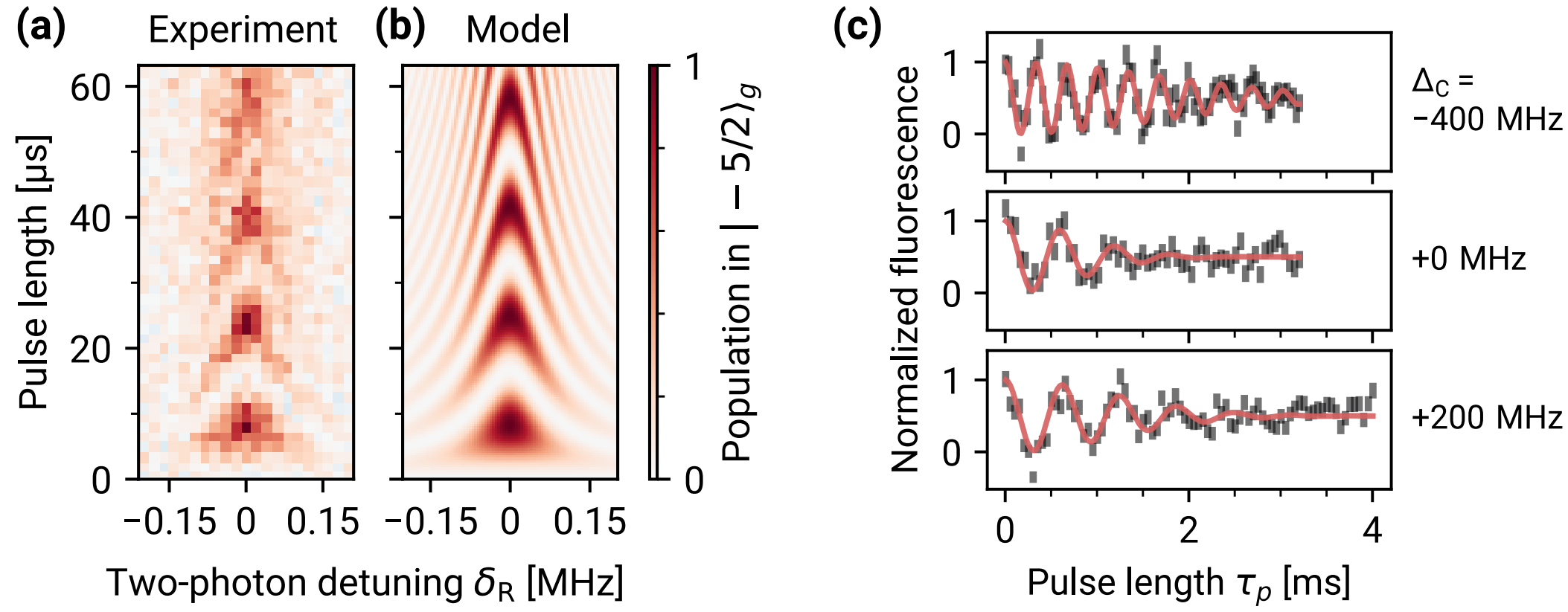

Supplementary Fig. S16. **Coherent population transfer between adjacent hyperfine states.** (a) Rabi oscillation on the hyperfine transition $|-7/2\rangle_g \leftrightarrow |-5/2\rangle_g$. The color scale represents the population in the upper ground state, $|-5/2\rangle_g$, after a Raman pulse ($\Delta = -120\,\text{MHz}$ and $\Delta_C = -600\,\text{MHz}$) with varying pulse length τ_p and two-photon detuning δ_R is applied. The ^{167}Er dopant is initially found in the lower ground state, $|-7/2\rangle_g$. (b) Expected population transfer probability, assuming an ideal Raman pulse without damping (Eq. 21). (c) Rabi oscillations on two-photon resonance for $\Delta = -90\,\text{MHz}$ and different cavity detunings Δ_C, yielding a Raman pulse fidelity (top to bottom) $\mathcal{F}_R = 0.98, 0.74, 0.86$. The fluorescence is probed on the $|-7/2\rangle_e \rightarrow |-7/2\rangle_g$ transition. Error bars: 1 SD.

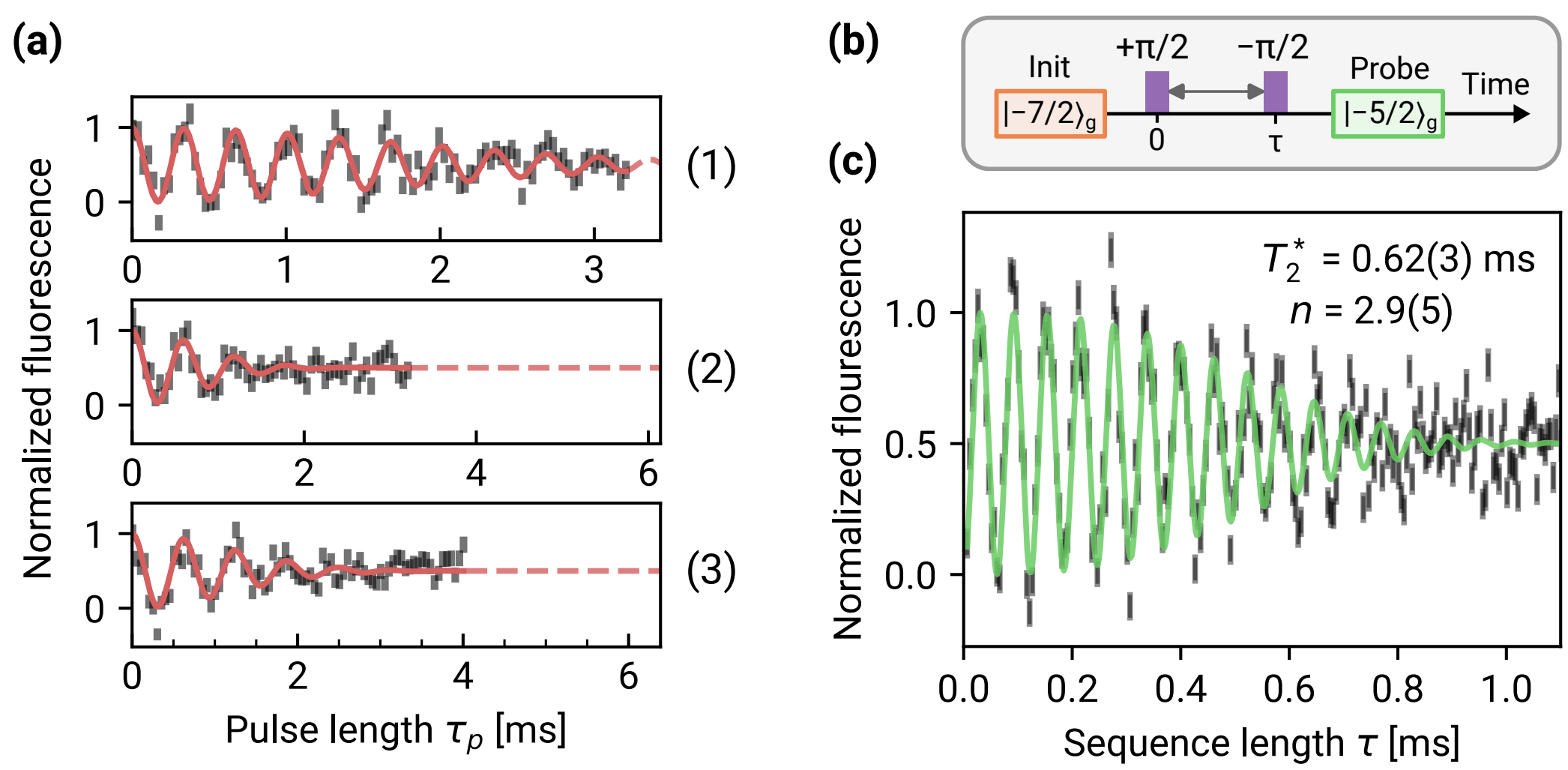

Supplementary Fig. S17. **Rabi oscillations and hyperfine dephasing of an ^{167}Er dopant.** (a) Rabi oscillations on the lowest hyperfine transition, $|-7/2\rangle_g \leftrightarrow |-5/2\rangle_g$, recorded at increasing Rabi frequencies from (1) to (3), while the cavity detuning is kept fixed at $\Delta_C = -400\,\text{MHz}$. Solid lines are fits to a damped oscillation with a stretched-exponential envelope ($n = 2$), yielding damping constants $T_d = 2.4(3), 0.36(4), 0.07(2)\,\text{ms}$, respectively. (b) Ramsey sequence with a $+\pi/2$ and $-\pi/2$ Raman pulse separated by a waiting time τ. Readout is performed on the $|-5/2\rangle_g$ state. (c) Normalized fluorescence (black bars) on the spin-preserving transition after the Ramsey sequence, as a function of τ, and for a constant two-photon detuning $\delta_R = 16.25(2)\,\text{kHz}$. The solid line is a fit to a damped oscillation with a stretched-exponential envelope; the fit parameters are indicated in the panel. Error bars: 1 SD.

with a stretched-exponential envelope. In all measurements (1-3), the observed decay is non-exponential with $n \approx 2$, suggesting that the dephasing results from time-correlated noise. We find the longest damping constant $T_d = 2.4(3)\,\text{ms}$, when the emitter is driven at the lowest Rabi frequency $\Omega_R = 2.97(1)\,\text{kHz}$, as shown in panel (1). When the Rabi frequency is increased, the pulse fidelity remains nearly unchanged with $\mathcal{F}_R = 0.97(1)$. However, at the same time, the decay constant T_d is reduced. Thus, in the panels (2) and (3), the predominant contribution to the damping can be attributed to amplitude fluctuations of the control fields.

To investigate the influence of emitter dephasing, we implemented a Ramsey sequence, consisting of two pulses ($+\pi/2$ and $-\pi/2$), separated by the time interval τ as shown in Fig. S17b. We use a detuning of $\delta_{\mathrm{R}} = 16.25(2)$ kHz and a Rabi frequency of $\Omega_{\mathrm{R}} = 78(1)$ kHz (equivalent to Fig. S17a, bottom panel), which corresponds to a pulse length $\tau_p = 3.5$ µs. The Raman pulses are performed with the same one-photon and cavity detuning as in Fig. S17a, panel (3). Compared to the Rabi oscillation with a damping constant $T_d = 0.07(2)$ ms, the Ramsey decay is much slower, yielding $T_2^* = 0.62(3)$ ms and $n = 2.9(5)$. As the duration of the Raman pulses is typically much shorter than T_2^*, emitter dephasing does not limit the observed Raman control pulse fidelity.

We thus conclude that fluctuations of the Raman pulse area cause the observed decay of the Rabi oscillations. A likely explanation is power fluctuations of the control laser that originate from imperfect preservation of the polarization state at fiber-fiber connectors between the polarization-maintaining components of the used fiber-optical setup. With typical polarization extinction ratios of 20 dB according to the data sheets of the manufacturers, the resulting power fluctuations of a few percent after several connectors explain the observed damping of the Rabi oscillations. In the future, this imperfection could be eliminated by an active stabilization of the pulse power. This will likely allow for a further improvement of the hyperfine coherence of single ^{167}Er emitters by dynamical decoupling, as described in the main text.

3. *Hahn-echo measurements*

As described in the main text, we use the sequence shown in Fig. S18a with pulse spacing τ and total length $T_{\mathrm{S}} := 2\tau$ for echo measurements on the hyperfine transition. The coherence is then extracted by subtracting the fluorescence measured during the readout on a spin-preserving transition for sequences with orthogonal phase ($+\pi/2$ and $-\pi/2$). Experimental results for the outermost hyperfine transitions are presented in Fig. S18 (b,c), for a magnetic field strength of $B = 6.8$ T. On the lowest hyperfine transition in the panel (b), $|-7/2\rangle_g \leftrightarrow |-5/2\rangle_g$, a coherence time of $T_{\mathrm{Hahn}} = 16.7(9)$ ms is extracted from a fit to a stretched-exponential function: $P_{\mathrm{Echo}}(\tau) = \frac{1}{2}\{\exp[-(2\tau/T_{\mathrm{Hahn}})^n] + 1\}$. In contrast, the upper hyperfine transition in panel (c), $|+5/2\rangle_g \leftrightarrow |+7/2\rangle_g$, exhibits a twofold longer coherence time $T_{\mathrm{Hahn}} = 32(1)$ ms. The chosen ^{167}Er dopant with an optical frequency at $\Delta^{|-7/2\rangle}_{|-7/2\rangle} = 195\,070.0$ GHz belongs to the outmost red-detuned satellite line. Hahn-echo measurements in the main text (Fig. 4c) were performed on a different dopant with an optical frequency at $\Delta^{|-7/2\rangle}_{|-7/2\rangle} = 195\,109.8$ GHz.

In the echo measurement in Fig. S18b, performed with 450 averages for each data point, one observes an initial collapse, followed by a partial revival of the coherence around $T_{\mathrm{S}} \approx 32$ ms. In the following, we will show that this can be explained by a slowly oscillating magnetic field (≈ 1.4 Hz) whose effect was already observed on the optical transitions. Due to a lower number of averages, a clear signature of this feature is, however, not observed in the other measurement (Fig. S18c).

To model the impact of the field modulation on the hyperfine coherence, we first estimate magnetic-field sensitivity using the crystal-field model [11]. Here, the sensitivity of the lowest hyperfine transition to magnetic field fluctuations is 10^3 to 2×10^3 lower than that of the respective spin-preserving optical transition. With a measured peak-to-peak variation of the optical transition frequency of ≈ 0.5 MHz, this yields a variation on the hyperfine transition of up to 0.5 kHz, when considering a perturbation in any of the three crystal axes (D1, D2, b).

We now consider a sinusoidal modulation of the two-photon detuning caused by an oscillating magnetic field, as shown in Fig. S19a, which follows $\delta'_{\mathrm{R}}(t) = \delta_{\mathrm{R}} + \delta_{\mathrm{mod}} \sin(2\pi\nu_{\mathrm{mod}} t + \phi_{\mathrm{mod}})$ with an arbitrary phase between each echo sequence and the modulation ϕ_{mod}. Here, the phase-averaged echo signal can be calculated as: [17]

$$\langle \hat{\sigma}_z \rangle(\tau) = J_0 \left[4 \frac{\delta_{\mathrm{mod}}}{\nu_{\mathrm{mod}}} \sin^2 \left(\pi \nu_{\mathrm{mod}} \tau \right) \right]. \tag{26}$$

Here, J_0 denotes the zero-order Bessel function of the first kind. The parameters δ_{mod} and ν_{mod} describe the modulation amplitude and frequency, which set an upper bound on the effective coherence time. Other decoherence processes are not included in this estimation.

For constant modulation parameters, the resulting coherence is plotted for $\delta_{\mathrm{mod}} = 0.5$ kHz and $\nu_{\mathrm{mod}} = 1.4$ Hz in Fig. S19b (gray line). One first observes a collapse of the coherence; later, it exhibits a full revival after 1.4 s. At shorter times (inset), partial revivals can be observed. This oscillation will be damped if the magnetic field noise exhibits several frequency components, or if its amplitude is randomly changing. To show this, we perform a numerical simulation in which we assume a Gaussian probability distribution $p(x) \sim \exp\left[-(x - \delta_{\mathrm{mod}})^2/(2\sigma^2)\right]$ with $\sigma = 0.1\delta_{\mathrm{mod}}$ (red line). This curve can be approximated by a stretched-exponential function (dashed blue line), in particular from $T_{\mathrm{S}} = 0$ until the first zero crossing. For large T_{S}, the approximation is only valid under the assumption of a large amplitude variation σ. When fitting the numerically calculated coherence decay function with $\sigma = 0.1\delta_{\mathrm{mod}}$, we obtain $T_{\mathrm{Hahn}} = 15.7(1)$ ms and $n = 6.0(4)$. For XY(N) decoupling sequences with N being the number of π pulses, numerical simulations yield a power-law scaling of $T_{\mathrm{DD}} \propto N^{0.84(4)}$ (Fig. S19c), in good agreement with the experimental results.

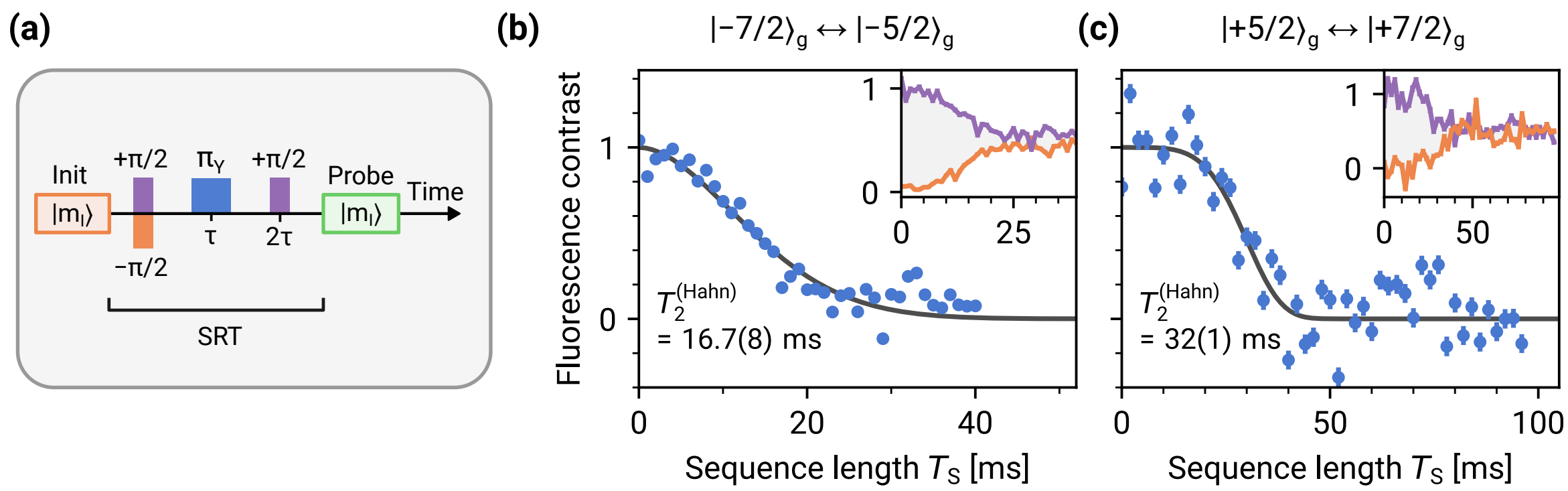

Supplementary Fig. S18. **Hahn-echo on ^{167}Er hyperfine transitions.** (a) Experimental sequence. After optical initialization, the Hahn-echo sequence is implemented by stimulated Raman transitions (SRT), and followed by optical readout. The duration of a Raman π pulse is 0.5 ms. (b,c) Normalized fluorescence (blue) as a function of the Hahn-echo sequence length $T_\mathrm{S} := 2\tau$ on the $|-7/2\rangle_g \leftrightarrow |-5/2\rangle_g$ (b) and $|+5/2\rangle_g \leftrightarrow |+7/2\rangle_g$ (c) hyperfine transitions. The data shown in the main panel (blue) is obtained by subtracting the fluorescence for a phase of $+\pi/2$ and $-\pi/2$ on the first Raman pulse (inset, orange and violet). Stretched-exponential fits (solid line) yield T_Hahn, as indicated in the panels, and $n = 1.7(2), 5(1)$ in (b,c), respectively. Error bars: 1 SD.

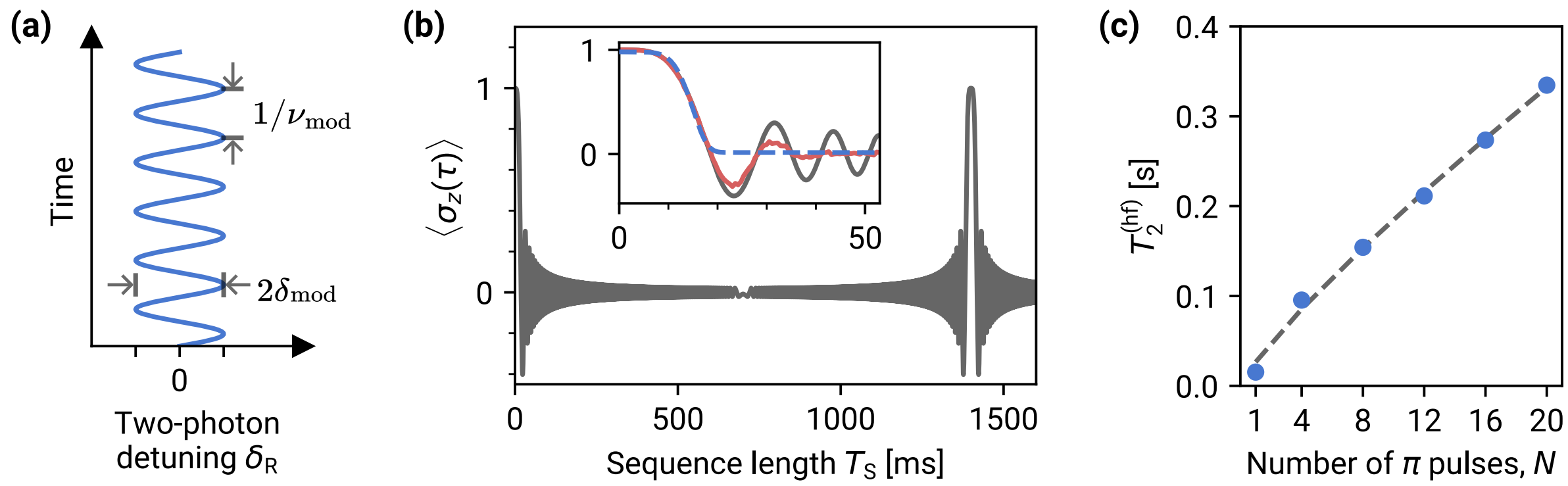

Supplementary Fig. S19. **Simulation of the spin coherence decay in an oscillating magnetic field.** (a) We assume a sinusoidal oscillation of the spin transition frequency in the time domain with amplitude δ_mod and frequency ν_mod. (b) Under these conditions, a drop of the spin coherence (i.e., the expectation value of the $\hat{\sigma}_z$ Pauli operator) is obtained when varying the sequence length $T_\mathrm{S} := 2\tau$. The gray line shows the coherence for a modulation with constant $\delta_\mathrm{mod} = 0.5$ kHz and $\nu_\mathrm{mod} = 1.4$ Hz. For a randomly changing amplitude with a Gaussian probability distribution ($\sigma = 0.1\delta_\mathrm{mod}$), the oscillation is further damped with increasing sequence length (red line). An approximation using a stretched-exponential function [$T_\mathrm{Hahn} = 15.7(1)$ ms and $n = 6.0(4)$] is shown as dashed blue line. (c) Numerically calculated coherence time for XY(N) decoupling sequences, yielding a power-law scaling $T_\mathrm{DD} \propto N^{0.84(4)}$ (dashed line).

The same fit function is used as an approximation for the experimental data. Here, the measured coherence on the uppermost hyperfine transition (Fig. S18c) exhibits a stretching factor $n = 5(1)$, which matches the simulated value within the fit uncertainty. The obtained coherence time is of a similar order of magnitude as the expectation; however, it is subject to a larger uncertainty. This is explained by the unknown direction of the magnetic-field perturbation and the inaccuracy when extrapolating the crystal-field model [11] to the studied parameter regime.

On the lowest hyperfine transition (Fig. S18b), a smaller stretching factor of $n = 1.7(2)$ is determined from the fit. This may suggest that a second mechanism is involved, such as magnetic-field noise from the nuclear spin bath. Still, the observed revival supports the hypothesis that the nuclear spin is also subject to a modulation of the magnetic field. The twofold shorter coherence time compared to the uppermost hyperfine transition disagrees with the crystal-field model, which predicts a lower sensitivity on this transition. Thus, this model may need further refinements.

4. Comparison to earlier works

Previous studies on the hyperfine coherence of an ^{167}Er ensemble in YSO were performed under different experimental conditions, investigating site 2 at $B = 7\,\mathrm{T}$ along the D1 axis and a temperature of $T = 1.4\,\mathrm{K}$). Under these conditions, a Hahn-echo coherence time of $T_2 = 1.30(1)\,\mathrm{s}$ was demonstrated [10], which exceeds the observed value by a factor of 40. Despite differing experimental parameters, in particular a different crystallographic site, a longer coherence time than 32(1) ms would also be expected in a stable magnetic field for site 1. Based on the crystal-field model [11], ^{167}Er dopants in site 1 exhibit only an approximately 3-fold higher magnetic field sensitivity under the experimental conditions in our work, suggesting the Hahn-echo coherence time may be extended up to $\approx 0.4\,\mathrm{s}$. In this regime, the primary origin of magnetic field noise limiting the coherence time likely stems from fluctuations in the nuclear spin bath. Due to the frozen core effect [18], however, only distant nuclear spins have a significant impact. Thus, an improvement in the stability of the external bias field is expected to further enhance the hyperfine coherence. Alternatively, a dynamical decoupling sequence can mitigate this kind of magnetic-field noise, as shown in the main text.

SUPPLEMENTARY REFERENCES

[1] B. Merkel, *Enhancing the Emission and Coherence of Erbium Dopants*, Ph.D. thesis, Technische Universität München (2021).
[2] A. Ulanowski, J. Früh, F. Salamon, A. Holzäpfel, and A. Reiserer, Adv. Opt. Mat. **12**, 2302897 (2024).
[3] N. C. Pisenti, A. Restelli, B. J. Reschovsky, D. S. Barker, and G. K. Campbell, Review of Scientific Instruments **87**, 124702 (2016).
[4] D. R. Leibrandt and J. Heidecker, Review of Scientific Instruments **86**, 123115 (2015).
[5] R. Kalra, A. Laucht, J. P. Dehollain, D. Bar, S. Freer, S. Simmons, J. T. Muhonen, and A. Morello, Review of Scientific Instruments **87**, 073905 (2016).
[6] N. L. Jobbitt, J.-P. R. Wells, M. F. Reid, S. P. Horvath, P. Goldner, and A. Ferrier, Phys. Rev. B **104**, 155121 (2021).
[7] A. Abragam and B. Bleaney, *Electron Paramagnetic Resonance of Transition Ions* (OUP Oxford, 2012).
[8] P. Raghavan, Atomic Data and Nuclear Data Tables **42**, 189 (1989).
[9] P. Goldner, A. Ferrier, and O. Guillot-Noël, in *Handbook on the Physics and Chemistry of Rare Earths*, Vol. 46, edited by J.-C. G. Bünzli and V. K. Pecharsky (Elsevier, 2015) pp. 1–78.
[10] M. Rančić, M. P. Hedges, R. L. Ahlefeldt, and M. J. Sellars, Nat. Phys. **14**, 50 (2018).
[11] S. Horvath, J. Rakonjac, Y.-H. Chen, J. Longdell, P. Goldner, J.-P. Wells, and M. Reid, Phys. Rev. Lett. **123**, 057401 (2019).
[12] A. Ulanowski, B. Merkel, and A. Reiserer, Sci. Adv. **8**, eabo4538 (2022).
[13] A. Ulanowski, O. Kuijpers, B. Merkel, A. Holzäpfel, and A. Reiserer, PRX Quantum **6**, 020344 (2025).
[14] M. Raha, S. Chen, C. M. Phenicie, S. Ourari, A. M. Dibos, and J. D. Thompson, Nat. Commun. **11**, 1605 (2020).
[15] A. Dibos, M. Raha, C. Phenicie, and J. Thompson, Phys. Rev. Lett. **120**, 243601 (2018).
[16] B. W. Shore, *Manipulating Quantum Structures Using Laser Pulses* (Cambridge University Press, Cambridge, 2011).
[17] A. Laraoui, J. S. Hodges, and C. A. Meriles, Appl. Phys. Lett. **97**, 143104 (2010).
[18] T. Böttger, C. W. Thiel, Y. Sun, and R. L. Cone, Phys. Rev. B **73**, 075101 (2006).
