# Supplementary material for: Cavity-enhanced optical readout and control of nuclear spin qubits
Source: Nat Commun. 2026 Aug 29;17:9234. doi: 10.1038/s41467-026-77060-8 (PMC13526025; doi:10.1038/s41467-026-77060-8)
Supplement: Supplementary file 2 [file 41467_2026_77060_MOESM2_ESM.pdf]

nature portfolio

Peer Review File

Cavity-enhanced optical readout and control of nuclear spin qubits

Corresponding Author: Professor Andreas Reiserer

This file contains all reviewer reports in order by version, followed by all author rebuttals in order by version.

Version 0:

Reviewer comments:

Reviewer #1

(Remarks to the Author)
The manuscript by A. Ulanowski and coauthors provides an experimental study of a single erbium dopant embedded in a YSO crystal and coupled to a tuneable microcavity. The narrow cavity linewidth allows the selective enhancement of a single optical transition and an improved optical cyclicity. This capability, in conjunction with an improved collection efficiency, allows a direct optical single-shot readout of the hyperfine state with 91% fidelity. The authors additionally use a Raman scheme to coherently couple two hyperfine ground states and to measure their coherence.

Using a cavity to improve the optical readout of an electronic spin is a well-established technique; however, its application to directly measuring the hyperfine state is, to my knowledge, novel. The nuclear spin is a particularly interesting qubit for quantum network applications requiring long coherence times. As such, this paper provides a nice approach for a nuclear spin-photon interface with coherent optical nuclear spin control.

Generally, the paper is well written and understandable. I find the methodology and the results convincing. The supplementary information will surely be of value to those wishing to recreate the experiment. There are a few places in the main text where knowledge specific to Erbium is required of the reader, and I believe a wider readership could be reached if more physical values are given in the main text. Overall, I recommend the article for publication, provided that the authors address the comments below.

=== Specific comments ===
1. To help the reader understand all relevant timescales of the experiment, I suggest the authors state the lifetime of the optically excited state in the absence and presence of Purcell enhancement. It is also not clear to me if the quoted enhancement of 95 (line 109) is measured or calculated.
2. When explaining spin flips due to optical decay (line 97), it would be easier to reference Fig. 1b than the supplementary information.
3. I don’t understand the statement on line 144 that spin-flipping transitions are not Purcell enhanced. As the laser and cavity are tuned in concert during the measurement, the non-spin-flipping transition should also be enhanced. Is there an enhancement difference due to the polarisation of the optical dipoles? For a non-expert, it is not clear how the optical dipoles are oriented relative to the cavity field.
4. Figure 1d shows the Purcell factor rapidly decaying away from resonance. While this follows the common definition of cavity Purcell enhancement, it should be noted that the total decay rate of non-resonant transitions is usually not significantly inhibited for an open cavity due to leaky radiation modes. I believe this could be made clearer from the paper. What matters for enhancing the optical cyclicity is the total decay rate and not just the decay rate into the cavity. In other words, it should be made clear that Fig. 1d only represents the contribution to the decay rate stemming from the cavity. One could otherwise draw the (I assume incorrect) conclusion that the cavity is enhancing the branching ratio by a factor 1000. To avoid confusion, it would also be useful to explicitly state the branching ratio of the I=-7/2 excited state with and without the cavity enhancement.
5. The nuclear spin lifetime stated on line 280 should have a citation or a figure reference.
6. Is excited state scattering currently limiting the Raman fidelity? This was not completely clear to me. I believe the scattering rate should be straight forward to calculate as the Purcell-enhanced excited state lifetime and optical detuning is known.
7. The Fig. 4c y-axis should be “contrast”. Fluorescence would tend towards 50% in an echo sequence as the spin state

becomes fully mixed.
8. What are the fit models being used in Fig. 4b and Fig. 4c? What is the dashed line in Fig. 4d? Please provide an explanation in the caption.
9. The echo revival around 30 ms in Fig. 4c hints at the presence of a neighbouring spin or two-level fluctuator. Can the authors comment on the likely origin of this feature?
10. I note the absence of references to recent works on Nuclear spin readout in silicon including [Phys. Rev. Lett. 132, 180803] and [Phys. Rev. Lett. 132, 180804]. While they rely on repetitive state transfer between the electron and nuclear spin, I believe they should be included as a reference to the state-of-the-art readout fidelity. Additionally, more related works on Er:YSO in microcavities could be referenced such as [Nat Commun 12, 3570 (2021)].

Reviewer #2

(Remarks to the Author)
The paper by A. Ulanowski et al. describes the all-optical initialization, manipulation and readout of the nuclear spin of a single 167Er ion in a thin Y2SiO5 crystal at a temperature < 1.8 K and a magnetic field of 6.8 T. A tunable high-finesse Fabry-Perot micro-cavity of bandwidth 65 MHz selectively Purcell-enhances the desired transition and increases the emission rate and collection efficiency. This allows the authors to use the m_I={-7⁄2,-5⁄2} manifold as qubit and demonstrate an initialization fidelity of 97.3 %, a single-shot readout fidelity of 91 % and a π-pulse fidelity up to 98 %. They furthermore measure the coherence through Hahn echo and dynamical decoupling, reaching up to 0.28 s coherence time.
We therefore think that the work contributes significantly to the progress towards a quantum network based on long-coherence-time spins connected through telecom wavelength photons. Given that the authors address the following points, we recommend the paper for publication in Nature Communications:
Line 42ff: It is not clear to us which electron-nuclear spin quantum gates are meant. Please add more details to the explanation and references in which these gates are used. Also, it is not clear to us why the authors say that “applying a large magnetic field overcomes the issues related to random electron spin flips”. Indeed, such random flips might still be possible upon relaxation from the optical excited state, or are we missing something?
Line 95: The paper only addresses the issue of preserving m_I. Please add some information as to whether m_s might be changed during the optical transitions.
Line 126: We would replace “tailor” with “decrease”, as it is more precise.
Line 131: “superconducting nanowire single-photon detectors” will avoid potential confusion with superconducting circuits designed to detect single microwave photons.
Line 146: The term spin pumping (also referred to as optical pumping in line 161) is only explained in the next section. Please re-order these.
Various questions which came to our mind while reading the manuscript are answered in the supplementary material. We would recommend referencing them in the main text, so that the interested reader can find them easily. A non-exhaustive list:
Line 165f: Branching ratio can be found in SI Fig. S4b.
Fig. 2: Reference to the SI section which describes how you find which peaks belong to the same spin.
Fig. 4: We were wondering if the resonator is brought back to resonance for the readout, until we found the answer in SI Fig. S14a.
Line 399: Outcoupling efficiency is calculated in SI Section 1B.
There are multiple general references to the supplement. We recommend referencing the corresponding section in the SI.
Fig. 1d: We are not sure if we understand the meaning of the colored areas. If it indicates the discrete transition frequencies corresponding to Δm_I={-1,0,+1}, it would be clearer with discrete lines instead of a color area. Otherwise, it could be misinterpreted as the inhomogeneous broadening.
Fig. 1 caption: Replace “up to 0.9 GHz” with “0.9 GHz at XX T magnetic field”.
Line 216: “we define a/the qubit”.
Fig. 2 would improve if the “arb. u.” could be replaced by a physically meaningful unit, such as “photons per pulse”.
Line 235ff: The Purcell filtering has been described in multiple other occasions prior to this paragraph. Please reduce redundancy where possible.
Line 253ff: The way the readout optimization is described is confusing. As shown in Fig. 3b, the optimal number of readout pulses depends on the photon number threshold. Yet, the text first describes the optimal number of pulses, then the photon number threshold.
Line 279ff: The lifetime measurement is neither supported by data nor discussed. Can the authors hypothesize on the possible mechanism for relaxation?
Line 297: Presumably “fluctuations” refer to the length of the cavity? This wording may induce confusions in the reader’s mind with respect to “quantum fluctuations”, so better to say “residual frequency fluctuations” or something similar.
Line 355: Does “optical scattering during the Raman pulses” mean that the excited state becomes populated due to the Raman drive and subsequently emits incoherent light?
Fig. 4: Does the described sequence have a name, such as XY8?
Line 367: Presumably, most of the power is reflected because it is detuned from the cavity.
Line 373: The sentence is written in an assertive way, but selective addressing is not proven here. So, the sentence should be rewritten to clearly express that it is a perspective.
References: We would find it more convenient if the references were cited with their title included.

SI section I E: The “second modulation” is mentioned before the first.
SI section II B1: What is meant with “satellite peak”?
SI Fig. S6: Making the blue trace partially transparent would improve the visibility of the orange trace.
SI section III: The wrong figure is referenced: “…as a function of N_init in Fig. S9b” should instead read “Fig. S8b”.

SI section V D: Instead of “detection bins” we would call it “time bins” for clarity.
SI section V D: “b ∈ N denoting the readout block length”.
SI section VI B: In all other occurrences in the paper, the energy decay rate is used instead of the field decay rate.
SI section VI B: Please mention the order of magnitude of the cavity vibrations.
SI section VI C2: The stretching factor n is used in an equation, but only named on the next page.
SI section VI C3: It is not clear to us how a slowly oscillating magnetic field can lead to an apparent revival of coherence.
SI references: Please provide DOI hyperlinks, as in the main text.

Reviewer #3

(Remarks to the Author)
I co-reviewed this manuscript with one of the reviewers who provided the listed reports. This is part of the Nature Communications initiative to facilitate training in peer review and to provide appropriate recognition for Early Career Researchers who co-review manuscripts.

Version 1:

Reviewer comments:

Reviewer #1

(Remarks to the Author)
Response to Author Rebuttal

I am generally satisfied with the authors’ response to my queries and the changes made to the manuscript.

A few issues however remain:
1)
The figure 4b caption states, “A cosine function with a stretched-exponential envelope (solid and dashed lines) fits the data well.” I only see a solid line. Either remove the reference to a dashed line or edit the figure appropriately.
2)
Is there a reason the errorbars in Fig. 4b looking different than in Fig. 4c? As a minor note, the errorbars are almost certainly overestimated as none of the datapoints deviate by more than one standard deviation. However, this does not alter the conclusion.
3)
I thank the authors for answering my question regarding the echo revival. However, carefully studying the supplementary material section on Hanh Echo has raised additional questions and some doubts about the analysis:
3a)
The authors claim that the revival is due to a magnetic field oscillating at 1.4 Hz. Equation (26) then predicts a revival at a sequence length 2\tau = 2/1.4Hz = 1.4 s. This does not match the observed revival at 28 ms. Going the other way, the 2\tau = 28 ms revival implies a frequency of 71 Hz.
3b)
According to the figure caption, the dashed line in Fig. S18b shows the coherence under a magnetic field modulated at constant frequency and amplitude. Apart from the wrong frequency (see previous point), the behaviour is incorrect. An echo sequence sensing only a delta-function noise source will perfectly rephase every time the half-duration is an integer number of noise periods, as also given by equation (26). The dashed line must include an additional noise source not explained in the text.
3c)
If the noise amplitude varies from shot-to-shot but is constant over a single echo sequence (i.e. it is quasistatic), the echo should STILL rephase perfectly as the rephasing condition only depends on the noise frequency. Thus, the behaviour of the red line in Fig. S18b does not make sense given the described model. For the magnetic field fluctuation to reproduce the observed coherence decay, you either need the noise to vary in frequency shot-to-shot (or be broadband) or the amplitude to vary during a single shot.
3d)
It would be helpful if the authors were consistent in denoting the sequence duration. Both T_S and 2\tau are used. I don’t think T_S is defined.
3e)
In the discussion, the authors rightfully assert that other noise mechanisms might be present, and that classical magnetic field noise cannot explain all experimental findings such as the varying stretching factor across different transitions. I would like to see this reservation reflected in the main text, which confidently pinpoints magnetic field noise as the sole cause. Adding one or two words should be enough to qualify the claims (the details are indeed more appropriate for the supplementary information).
4)
The supplementary information on Raman control states, “Thus, we implement coherent control via optical Raman fields, detuned by ≈ 0.9 GHz”. To me, this implies a one-photon detuning, yet the meaning must be that the two field components are split by 0.9 GHz in accordance with the ground state splitting.
5)
In the supplementary section on Raman pulse fidelity, the authors state “The damping constant Td hereby corresponds to the lower bound of the emitter dephasing time T_2^*.” This statement is wrong. Driving a qubit protects it against dephasing

from T_2*. In the limit of Omega tending to infinity, the grand Rabi frequency no longer depends on the two-photon detuning, and the oscillations continue forever. A nice experimental example of protecting a solid-state spin through driving can be found in [Bodey, J.H., Stockill, R., Denning, E.V. et al. Optical spin locking of a solid-state qubit. npj Quantum Inf 5, 95 (2019).]

I also agree with the other reviewer that the presentation is lengthy and could be made more concise by cutting down on repetitions. A few examples:

1.
Too much information is repeated in the figure captions. This is especially true for the Fig. 1a caption, which repeats the motivation of the large magnetic field rather than concisely describing the elements of the figure.

2.
Similarly, the concept and limitations of qubit readout through photon counting is verbosely explained in both the main text and the caption of Fig. 3. Given how well established this technique is, the treatment could be shortened.

3.
The textbook theory treatment of Raman control in the supplementary information is not necessary. It is sufficient to quote the main results for a Lambda system by providing a reference. I find it odd that the authors include atomic recoil in the two-photon detuning (Supplementary equation 19) when it clearly doesn't apply to the studied system. Please don't add irrelevant information.

These suggestions are not crucial towards accepting the paper, but would, in my opinion, greatly improve the readability.

While the manuscript has been improved, I cannot recommend publication until my objections on the Hanh Echo analysis have been addressed.

Reviewer #2

(Remarks to the Author)
I am satisfied with the revised manuscript and recommend publication

Reviewer #3

(Remarks to the Author)
I co-reviewed this manuscript with one of the reviewers who provided the listed reports. This is part of the Nature Communications initiative to facilitate training in peer review and to provide appropriate recognition for Early Career Researchers who co-review manuscripts.

Version 2:

Reviewer comments:

Reviewer #1

(Remarks to the Author)
I thank the authors for their work in improving the manuscript and answering my queries.

I had indeed misunderstood the analysis of the partial spin echo revival in the supplementary material. Thanks to the updated figures and text in the new version, the analysis is now clear and unambiguous.

I fully recommend this paper for publication and again congratulate the authors on their nice results.

Open Access This Peer Review File is licensed under a Creative Commons Attribution 4.0 International License, which permits use, sharing, adaptation, distribution and reproduction in any medium or format, as long as you give appropriate credit to the original author(s) and the source, provide a link to the Creative Commons license, and indicate if changes were made.
In cases where reviewers are anonymous, credit should be given to 'Anonymous Referee' and the source.
The images or other third party material in this Peer Review File are included in the article's Creative Commons license, unless indicated otherwise in a credit line to the material. If material is not included in the article's Creative Commons license and your intended use is not permitted by statutory regulation or exceeds the permitted use, you will need to obtain permission directly from the copyright holder.
To view a copy of this license, visit https://creativecommons.org/licenses/by/4.0/

Reply to the reports on „Cavity-enhanced optical readout and control of nuclear spin qubits“

We thank both reviewers for their positive evaluation of our work and their detailed feedback, which has helped us to improve the manuscript further. In the following, we will provide a detailed response to their comments. All changes to the manuscript are marked in red. Line and Section references used below refer to the updated manuscript.

In addition to these changes, we updated the open data statement. With these changes, we are confident that the manuscript can be accepted for publication in Nature Communications.

Reviewer #1 (Remarks to the Author):

The manuscript by A. Ulanowski and coauthors provides an experimental study of a single erbium dopant embedded in a YSO crystal and coupled to a tuneable microcavity. The narrow cavity linewidth allows the selective enhancement of a single optical transition and an improved optical cyclicity. This capability, in conjunction with an improved collection efficiency, allows a direct optical single-shot readout of the hyperfine state with 91% fidelity. The authors additionally use a Raman scheme to coherently couple two hyperfine ground states and to measure their coherence.

Using a cavity to improve the optical readout of an electronic spin is a well-established technique; however, its application to directly measuring the hyperfine state is, to my knowledge, novel. The nuclear spin is a particularly interesting qubit for quantum network applications requiring long coherence times. As such, this paper provides a nice approach for a nuclear spin-photon interface with coherent optical nuclear spin control.

Generally, the paper is well written and understandable. I find the methodology and the results convincing. The supplementary information will surely be of value to those wishing to recreate the experiment. There are a few places in the main text where knowledge specific to Erbium is required of the reader, and I believe a wider readership could be reached if more physical values are given in the main text. Overall, I recommend the article for publication, provided that the authors address the comments below.

We thank the referee for the accurate summary of our work. We have added additional information about the investigated material platform to better target a broader audience, following the comments below.

=== Specific comments ===

1. To help the reader understand all relevant timescales of the experiment, I suggest the authors state the lifetime of the optically excited state in the absence and presence of Purcell enhancement. It is also not clear to me if the quoted enhancement of 95 (line 109) is measured or calculated.

To clarify, we added: “leads to a faster optical decay, reducing the excited-state lifetime of 11.4 ms [...] down to 0.12 ms.”, and “measured Purcell factor [...] of up to 95(10)”.

2. When explaining spin flips due to optical decay (line 97), it would be easier to reference Fig. 1b than the supplementary information.

As suggested, we have included a reference to Fig. 1b which shows the qualitative picture of the optical spin flips: "... when decaying optically on the Y1 <-> Z1 transition (see supplement), as indicated by the blue and red dotted arrows in Fig. 1b". We keep the reference to the Supplementary Information as it contains a more detailed and quantitative analysis based on the known spin Hamiltonian of ^{167}Er in site 1 in YSO.

3. I don't understand the statement on line 144 that spin-flipping transitions are not Purcell enhanced. As the laser and cavity are tuned in concert during the measurement, the non-spin-flipping transition should also be enhanced. Is there an enhancement difference due to the polarisation of the optical dipoles? For a non-expert, it is not clear how the optical dipoles are oriented relative to the cavity field.

Due to the low symmetry of the host crystal, the optical dipoles are not isotropic, and their precise value is still unknown. However, one can still make a statement about the Purcell enhancement, as the relative transition rates can be calculated from the spin Hamiltonian via the branching ratio β. Specifically, the effective Purcell enhancement P of an optical transition is $P = \beta P_{TL}$, where P_{TL} is the Purcell factor for a two-level system. As discussed in SI Sec. II and in particular Fig. S4b, the branching ratio of the spin-preserving transitions can be up to nine times higher than that of the spin-flip transitions. To clarify, we added: "The spin-flip transitions, which are detuned by a few hundred MHz, do not give a measurable signal (not shown) because their Purcell enhancement is reduced in proportion to the branching ratio (see Supplementary Information Sec. II A) [...]"

4. Figure 1d shows the Purcell factor rapidly decaying away from resonance. While this follows the common definition of cavity Purcell enhancement, it should be noted that the total decay rate of non-resonant transitions is usually not significantly inhibited for an open cavity due to leaky radiation modes. I believe this could be made clearer from the paper. What matters for enhancing the optical cyclicity is the total decay rate and not just the decay rate into the cavity. In other words, it should be made clear that Fig. 1d only represents the contribution to the decay rate stemming from the cavity. One could otherwise draw the (I assume incorrect) conclusion that the cavity is enhancing the branching ratio by a factor 1000. To avoid confusion, it would also be useful to explicitly state the branching ratio of the I=-7/2 excited state with and without the cavity enhancement.

We agree with the referee's comment that the total decay rate is the key figure of merit, and the inhibition of the decay is not significant in our cavity geometry. In the caption of Fig. 2b we already state that "using an optical resonator, the nuclear-spin-preserving optical transitions (green arrows) can be enhanced selectively, without altering the decays that increase (red dotted) or reduce (blue dotted) m_I by one quantum". To make this clearer we adapted the caption of Fig. 1d as follows: "Via the Purcell effect, the decay rate into the cavity mode on the transition $-7/2_e$ -> $-7/2_g$ (green line) is enhanced up to a factor of P=95(10), while detuned optical transitions can exhibit several orders of magnitude lower relative Purcell factors P/P_{max}." In addition, we added a sentence to the main text:

Thus, as shown in Fig. 1d, the decay rate of the detuned spin-flip transitions exhibits no enhancement as P << 1; it is thus unchanged from its bulk value. In contrast, the cavity selectively enhances the resonant nuclear-spin-preserving transitions, with a measured Purcell factor of up to 95(10). The resulting increase in cyclicity paves the way for all-optical nuclear-spin qubit readout demonstrated in this work.

5. The nuclear spin lifetime stated on line 280 should have a citation or a figure reference.

We added: "We start by measuring the lifetime, which is 33(3)s at the used temperature [...] and magnetic field [...] (see Supplementary Information Sec. II D)."

6. Is excited state scattering currently limiting the Raman fidelity? This was not completely clear to me. I believe the scattering rate should be straight forward to calculate as the Purcell-enhanced excited state lifetime and optical detuning is known.

As mentioned in the main text, "The oscillations are damped because of power and/or polarization fluctuations of the Raman laser pulses". To further clarify, we added: "To avoid that scattering from the excited state limits the spin control fidelity, [...]". It is further detailed in SI Sec. VI C 3 that excited state scattering is not a limiting factor.

7. The Fig. 4c y-axis should be "contrast". Fluorescence would tend towards 50% in an echo sequence as the spin state becomes fully mixed.

We changed the axis label to "fluorescence contrast" and adapted Fig. S19 accordingly as well.

8. What are the fit models being used in Fig. 4b and Fig. 4c? What is the dashed line in Fig. 4d? Please provide an explanation in the caption.

We added the models and explanation to the text as follows:

Fig. 4b: "A cosine function with a stretched-exponential envelope (solid line) fits the data well". The fitting function is already given in the Supplement.

Fig. 4c: "is obtained from a fit to a stretched-exponential function (solid line)". We added the corresponding function to the Supplementary Information.

Fig. 4d: "The dotted gray line shows the expected increase for a slowly varying spin bath, $\propto N^{2/3}$". Furthermore, we added an explanatory sentence to the text "in good agreement with the expectation from the fluctuating magnetic field in the setup, and different from the power law $\propto N^{2/3}$ expected for a slowly varying spin bath (see Supplementary Information)".

9. The echo revival around 30 ms in Fig. 4c hints at the presence of a neighbouring spin or two-level fluctuator. Can the authors comment on the likely origin of this feature?

We added: This reduction, and the revival of T_{Hahn} around 30 ms, originate from fluctuations of the bias magnetic field that can be reduced in the future by active damping (see Supplementary Information).

10. I note the absence of references to recent works on Nuclear spin readout in silicon including [Phys. Rev. Lett. 132, 180803] and [Phys. Rev. Lett. 132, 180804]. While they rely on repetitive state transfer between the electron and nuclear spin, I believe they should be included as a reference to the state-of-the-art readout fidelity. Additionally, more related works on Er:YSO in microcavities could be referenced such as [Nat Commun 12, 3570 (2021)].

We added the mentioned references to the introduction. They have been performed in silicon carbide. The reported not-post-selected readout fidelity of 95% and 92% is comparable to our combined readout and initialization fidelity of 91(2)%. Therefore, we did not make any further changes to the text.

The work presented in Nat Commun 12, 3570 (2021) differs from ours in the use of Er-doped Y_2O_3 in the form of nanoparticles. In this approach, additional scattering loss resulted in significantly lower Q-factors (by two orders of magnitude), making it unsuitable for the optical initialization and readout of the ^{167}Er nuclear spin. However, it may be achieved in improved devices; we therefore added a corresponding reference, and a reference on Er:Si in a high-Q Fabry-Perot resonator, to the outlook.

Reviewer #2 (Remarks to the Author):

The paper by A. Ulanowski et al. describes the all-optical initialization, manipulation and readout of the nuclear spin of a single 167Er ion in a thin Y2SiO5 crystal at a temperature < 1.8 K and a magnetic field of 6.8 T. A tunable high-finesse Fabry-Perot micro-cavity of bandwidth 65 MHz selectively Purcell-enhances the desired transition and increases the emission rate and collection efficiency. This allows the authors to use the m_I={-7⁄2,-5⁄2} manifold as qubit and demonstrate an initialization fidelity of 97.3 %, a single-shot readout fidelity of 91 % and a π-pulse fidelity up to 98 %. They furthermore measure the coherence through Hahn echo and dynamical decoupling, reaching up to 0.28 s coherence time.
We therefore think that the work contributes significantly to the progress towards a quantum network based on long-coherence-time spins connected through telecom wavelength photons. Given that the authors address the following points, we recommend the paper for publication in Nature Communications:

We thank referee #2 and #3 for the accurate summary, and their positive recommendation of our work. In the following, we address their comments point-by-point.

Line 42ff: It is not clear to us which electron-nuclear spin quantum gates are meant. Please add more details to the explanation and references in which these gates are used.

To clarify, we added: "and the indirect nuclear-spin readout via the electronic spin ancilla requires additional electron-nuclear spin quantum gates [Dutt 2007, Neumann 2010, Robledo 2011, Lai 2024, Hesselmeier 2024, Abobeih 2019] that are typically implemented by microwave pulses [...]"

Also, it is not clear to us why the authors say that "applying a large magnetic field overcomes the issues related to random electron spin flips". Indeed, such random flips might still be possible upon relaxation from the optical excited state, or are we missing something?

At the applied large magnetic field of 6.8 T, all electronic spins of Er and other impurities in the material are frozen in their ground state at 1.7 K. In principle, optical excitation can flip the electronic spin of the addressed dopant; however, this decay is not enhanced by the optical resonator, and thus much weaker than the electron-spin-preserving decays. Equally important, the electronic spin would immediately return back to thermal equilibrium due to fast phononic relaxation with a relaxation rate that scales with B^5. To clarify the latter aspect, we added the following sentence to the introduction: "When the induced Zeeman splitting strongly exceeds the thermal energy, the electronic spins are frozen to the ground state and decay to it on very fast timescales via the direct spin-lattice relaxation process [Wolfowicz 2021]. Still, it has been shown that hyperfine interaction can enable all-optical control of nuclear spin ensembles [Rancic 2018]."

Line 95: The paper only addresses the issue of preserving m_I. Please add some information as to whether m_s might be changed during the optical transitions.

We added: "Note that the effective S=1/2 electronic spin may decay optically to m_S=1/2, but in this case returns immediately to the thermal equilibrium state -1/2 because of direct spin-lattice relaxation at a rate proportional to B^5 [Wolfowicz 2021]."

Line 126: We would replace "tailor" with "decrease", as it is more precise.

We changed the sentence to: "Co-doping with Eu allows for a tailored decrease of the spectral density"

Line 131: "superconducting nanowire single-photon detectors" will avoid potential confusion with superconducting circuits designed to detect single microwave photons.

We changed it to "superconducting nanowire single-photon detectors".

Line 146: The term spin pumping (also referred to as optical pumping in line 161) is only explained in the next section. Please re-order these.

We have shifted the definition of optical pumping to line 98: "optical pumping, i.e., repeated excitation, ...". In addition, we have changed the respective sentence to improve clarity: "In addition, in the case of repeated probing at the same frequency, the signal will decay because of unintended optical spin pumping."

Various questions which came to our mind while reading the manuscript are answered in the supplementary material. We would recommend referencing them in the main text, so that the interested reader can find them easily. A non-exhaustive list:

Line 165f: Branching ratio can be found in SI Fig. S4b.

We added a reference to SI Sec. II A.

Fig. 2: Reference to the SI section which describes how you find which peaks belong to the same spin.

We added a reference to SI Sec. II C.

Fig. 4: We were wondering if the resonator is brought back to resonance for the readout, until we found the answer in SI Fig. S14a.

We now point out that the resonator "is temporarily detuned".

Line 399: Outcoupling efficiency is calculated in SI Section 1B.

We added a reference to SI Sec. I B.

There are multiple general references to the supplement. We recommend referencing the corresponding section in the SI.

We follow the referee's suggestion and now mention the corresponding sections wherever referring to the supporting information.

Fig. 1d: We are not sure if we understand the meaning of the colored areas. If it indicates the discrete transition frequencies corresponding to $\Delta m_I=\{-1,0,+1\}$, it would be clearer with discrete lines instead of a color area. Otherwise, it could be misinterpreted as the inhomogeneous broadening.

To clarify, we have adapted the caption: "The colored areas indicate the spectral bands containing spin-preserving (light green) and the spin-flip (blue and red) transitions."

During the preparation of the manuscript, we have tried to indicate them with vertical solid lines. However, some lines would merge for certain transitions with $\Delta m_I = 0$ and $\Delta m_I = +1$ (see Fig. S5, circle and triangle markers). This would result in less than 7 and 8 visible lines for the $\Delta m_I = +/-1$ and $\Delta m_I = 0$ transitions, respectively, which we found to be misleading. Therefore, we decided to indicate the bands as colored areas and the corresponding labels (-1, 0, +1) in the panel.

Fig. 1 caption: Replace "up to 0.9 GHz" with "0.9 GHz at XX T magnetic field".

In the main text we state the magnetic-field configuration: "When applying 6.8 T along the b-axis of the crystal, the nuclear spin levels are separated by up to 0.9 GHz [...]". Therefore, we changed the caption as follows: "separated by up to 0.9 T at the applied magnetic field".

Line 216: "we define a/the qubit".

We changed this: "we define the qubit".

Fig. 2 would improve if the "arb. u." could be replaced by a physically meaningful unit, such as "photons per pulse".

As mentioned in SI Sec. I F, the signal-to-noise ratio of the used single-photon detector depends on the set bias voltage, and is not optimal at the maximum quantum efficiency. While it is paramount for the single-shot readout to use the optimal SNR, it is not so critical for the fluorescence spectra. Therefore, they have been recorded at different detector efficiency.

To avoid confusing the reader by showing different detection efficiencies in different parts of the manuscript, we decided to normalize fluorescence intensities in measurements where absolute efficiency is irrelevant for the interpretation of the data. Thus, we prefer to keep arb. u. as the axis label. The maximum "photon detection efficiency [of up] to 11(1) %" achieved in our setup is stated in the main text for the interested reader.

Line 235ff: The Purcell filtering has been described in multiple other occasions prior to this paragraph. Please reduce redundancy where possible.

We shortened the corresponding paragraph to reduce redundancy.

Line 253ff: The way the readout optimization is described is confusing. As shown in Fig. 3b, the optimal number of readout pulses depends on the photon number threshold. Yet, the text first describes the optimal number of pulses, then the photon number threshold.

To avoid confusion, we now first show the histograms, use them to explain the discrimination threshold, and only then explain Fig. 3b that shows the systematic parameter optimization. To explain this, we added a new sentence: The histograms in Fig.3a were measured at the optimal pulse number and discrimination threshold. This is the result of a systematic optimization, shown in Fig.3b. From the histograms, it can be seen that [...]"

Line 279ff: The lifetime measurement is neither supported by data nor discussed. Can the authors hypothesize on the possible mechanism for relaxation?

We have added a new section in the Supplementary information (Sec. II D), which shows the data from which we obtained the lifetime of 33(3) s in our experimental configuration (T = 1.7K, B = 6.8 T). The relaxation mechanisms will require further investigations.

Line 297: Presumably "fluctuations" refer to the length of the cavity? This wording may induce confusions in the reader's mind with respect to "quantum fluctuations", so better to say "residual frequency fluctuations" or something similar.

We changed "fluctuations" to "residual frequency fluctuations".

Line 355: Does "optical scattering during the Raman pulses" mean that the excited state becomes populated due to the Raman drive and subsequently emits incoherent light?

To clarify, we changed the sentence to: "However, a limit may be reached because of the small probability of spontaneous decay after populating the optically excited state during the Raman pulses. This may hinder [...]"

Fig. 4: Does the described sequence have a name, such as XY8?

We now include the commonly used name in the main text: "the phase of the pulses is altered between rotations around the *X* and *Y* axes, forming an XY(*N*) sequence". In addition, we added a corresponding sentence to the SI: "For XY(*N*) decoupling sequences with *N* being the number of π pulses, numerical simulations yield a power-law scaling [...], in good agreement with the experimental results."

Line 367: Presumably, most of the power is reflected because it is detuned from the cavity.

We added: "is reflected because of the detuning from the cavity resonance"

Line 373: The sentence is written in an assertive way, but selective addressing is not proven here. So, the sentence should be rewritten to clearly express that it is a perspective.

We changed this sentence to "..., the technique could enable selective addressing".

References: We would find it more convenient if the references were cited with their title included.

We note this suggestion and will aim to provide the titles in future manuscript submissions.

SI section I E: The "second modulation" is mentioned before the first.

We thank the referees for pointing this out and have rewritten this paragraph such that $\nu_L(t)$ is termed as the "modulation" and the "secondary modulation" is used for the PDH scheme.

SI section II B1: What is meant with "satellite peak"?

For consistency, we changed all occurrences of "satellite peak" to "satellite line" and added an explanation on the first occurrence of this term: "satellite lines in the inhomogeneous distribution

that originate from europium co-dopants [Ulanowski 2024]”. The added reference further clarifies the definition.

SI Fig. S6: Making the blue trace partially transparent would improve the visibility of the orange trace.

The blue trace in Fig. S6d was already slightly transparent. To improve visibility, we have increased transparency further.

SI section III: The wrong figure is referenced: “…as a function of N_init in Fig. S9b” should instead read “Fig. S8b”.

We corrected the reference to Fig. S8b.

SI section V D: Instead of “detection bins” we would call it “time bins” for clarity.

We followed the suggestion and changed it to “time bins”.

SI section V D: “b∈N denoting the readout block length”.

To clarify, we added that b is the readout block index.

SI section VI B: In all other occurrences in the paper, the energy decay rate is used instead of the field decay rate.

We assume this comment refers to Eq. 22, where we have defined κ as the field decay rate. The factor 1/2π is added to obtain the natural frequency to match the unit of the detuning Δ. Also, the other occurrences, such as in Sec. I B, define κ as the field decay rate. We therefore do not see any inconsistency. However, at one instance in the SI, κ has not been clearly defined. We therefore added: “The field decay rate of the resonator κ = 2π 33(3) MHz is determined from the linewidth of the fundamental (00) mode”

SI section VI B: Please mention the order of magnitude of the cavity vibrations.

We have added the room-mean-square cavity frequency fluctuations to the supplement at the end of Sec. I E: “up to 23(7) MHz, which corresponds to approximately one third of the cavity linewidth (FWHM)”, and a reference in Sec. VI B.

SI section VI C2: The stretching factor n is used in an equation, but only named on the next page.

In the first paragraph of SI section VI C2, we now explicitly define the stretching factor n.

SI section VI C3: It is not clear to us how a slowly oscillating magnetic field can lead to an apparent revival of coherence.

As an example, one can consider AC magnetic field sensing with spin qubits, e.g. NV centers, as described in Ref. [Laraoui et al, 2010]. Here, a Hahn-Echo or DD sequence effectively acts as a frequency filter depending on the chosen inter-pulse spacing. In our case, due to the nuclear Zeeman effect, the transition frequency between two hyperfine levels is shifted proportionally to the change of the external field, while the driving frequency (the detuning between the two Raman fields) is kept at a constant frequency. For a Hahn-Echo with a single π-pulse, the accumulated phase shift can be fully reversed if the pulse separation matches exactly half the modulation period, leading to a full revival. When the modulation amplitude is large enough, the accumulated phase shift can exceed 2π, resulting in additional partial revivals. One such revival we observe in our data, e.g. Fig. 4c at ~30 ms.

Analytically, the envelope of the echo signal assuming an infinite T_2 time is described by Eq. 26, which we have adapted from Ref. [Laraoui et al, 2010] and plotted in Fig. S18b (dashed line). With this figure, the derivation and the reference, we think that the model is sufficiently introduced, such that we have not made any further changes to the corresponding paragraph.

SI references: Please provide DOI hyperlinks, as in the main text.

We have added hyperlinks to the references in the Supplementary Information.

Reviewer #3 (Remarks to the Author):

I co-reviewed this manuscript with one of the reviewers who provided the listed reports. This is part of the Nature Communications initiative to facilitate training in peer review and to provide appropriate recognition for Early Career Researchers who co-review manuscripts.

Reply to the reports on „Cavity-enhanced optical readout and control of nuclear spin qubits“

We thank both reviewers for their feedback on our revised manuscript. To address the raised concerns, we have made changes to the manuscript (*text in italics*, changes marked in red), and below, we provide a detailed response to their comments. In the Supplementary Information, we have swapped Figs. S18 and S19 to match the order of appearance in the main text. Additionally, we have added a DOI reference in the data availability section, which will be made publicly accessible at a later stage. With this, we look forward to the next steps towards publication.

Reviewer #1 (Remarks to the Author):

I am generally satisfied with the authors' response to my queries and the changes made to the manuscript. A few issues however remain:

1) The figure 4b caption states, "A cosine function with a stretched-exponential envelope (solid and dashed lines) fits the data well." I only see a solid line. Either remove the reference to a dashed line or edit the figure appropriately.

We thank the referee for pointing out that the caption was not updated in the resubmission. We now removed the reference to the dashed line.

2) Is there a reason the errorbars in Fig. 4b looking different than in Fig. 4c? As a minor note, the errorbars are almost certainly overestimated as none of the datapoints deviate by more than one standard deviation. However, this does not alter the conclusion.

The different styles of markers and errorbars in Fig. 4b did not have a physical meaning. For consistency, we changed the marker style to match the other panels. We also corrected the error bars which were showing 2 S.D. instead of one. We thank the referee for pointing this out. The same correction was applied to Fig. S16c and S17a.

3) I thank the authors for answering my question regarding the echo revival. However, carefully studying the supplementary material section on Hanh Echo has raised additional questions and some doubts about the analysis:

3a) The authors claim that the revival is due to a magnetic field oscillating at 1.4 Hz. Equation (26) then predicts a revival at a sequence length 2\tau = 2/1.4Hz = 1.4 s. This does not match the observed revival at 28 ms. Going the other way, the 2\tau = 28 ms revival implies a frequency of 71 Hz. 3b) According to the figure caption, the dashed line in Fig. S18b shows the coherence under a magnetic field modulated at constant frequency and amplitude. Apart from the wrong frequency (see previous point), the behaviour is incorrect. An echo sequence sensing only a delta-function noise source will perfectly rephase every time the half-duration is an integer number of noise periods, as also given by equation (26). The dashed line must include an additional noise source not explained in the text.

We will give a joint answer to comments 3a) and 3b) which are closely related. As correctly stated by the referee, when considering magnetic field noise at a frequency of 1.4 Hz, as done in our analysis, one would expect a full revival after 1.4 s. Therefore, as correctly stated by the referee (in 3a), the observed feature at 28 ms cannot and does not correspond to a full revival.

Instead, at shorter times, one can observe partial revivals. For suited parameters of the vibration amplitude, the measured initial decay and revival in Fig. S19 are well-captured by the analytical formula of Ref [18] (Laraoui et al. Appl. Phys. Lett. 97, 2010), just assuming a 1.4 Hz frequency modulation (i.e. a δ-function in frequency space, as suggested in question 3b).

In other words, while perfect rephasing will only occur when the half-duration is an integer number of noise periods, as stated by the referee, partial revivals are observed before that. To make this clear to the readers, we now show a larger temporal range in a new panel of Fig. S19b (using a solid gray line instead of the dashed line used earlier):

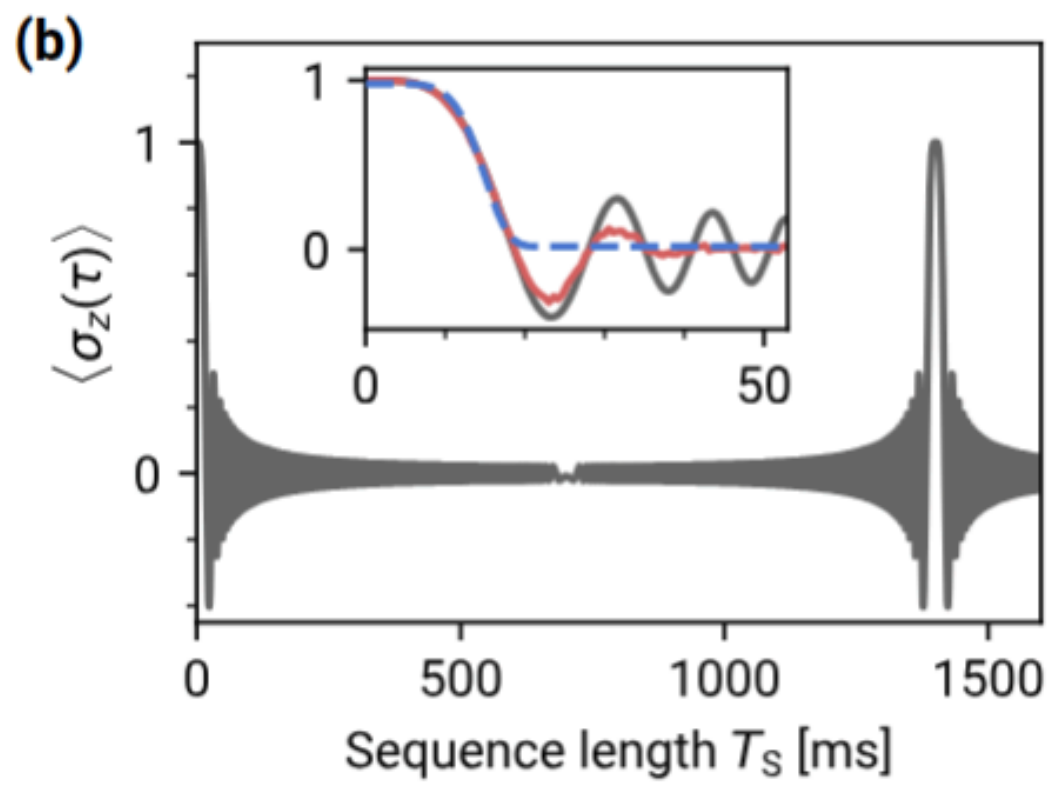

Here, one can clearly see the expected full revival at 1.4s; in addition, the graph shows the earlier, partial revivals in the time range up to 50 ms. In the submitted version of S19b, we have only shown this time range, potentially causing confusion. This is now avoided by showing the original panel as a zoom-in insert of S19b. Here, the grey line is the theory curve using the model of [18], with only 1.4 Hz noise and without any other noise sources.

Compared to the theory model, the measurements also exhibit a decay of the first partial revivals. This can be explained by additional frequency components or by fluctuations in the magnetic field noise amplitude, as suggested in the caption.

To further clarify this, we modified the text in the SI: "*In the echo measurement in Fig. S18b, performed with 450 averages for each data point, one observes an initial collapse, followed by a partial revival of the coherence around T_S ~ 32 ms. In the following, we will show that this can be explained by a slowly oscillating magnetic field (at ~1.4 Hz). [...]*

We now consider a sinusoidal modulation of the two-photon detuning caused by an oscillating magnetic field, as shown in Fig. S18a, which follows [...]

One first observes a collapse of the coherence; later, it exhibits a full revival after 1.4s. At shorter times (inset), partial revivals can be observed. This oscillation will be damped if the magnetic field noise exhibits several frequency components, or if its amplitude is randomly changing. To show this, we perform a numerical simulation in which we assume a Gaussian probability distribution...".

3c) If the noise amplitude varies from shot-to-shot but is constant over a single echo sequence (i.e. it is quasistatic), the echo should STILL rephase perfectly as the rephasing condition only depends on the noise frequency. Thus, the behaviour of the red line in Fig. S18b does not make sense given the described model. For the magnetic field fluctuation to reproduce the observed coherence decay, you either need the noise to vary in frequency shot-to-shot (or be broadband) or the amplitude to vary during a single shot.

Indeed, if magnetic field noise with a precise frequency of 1.4 Hz was the only noise source, one would expect a perfect revival after 1.4 s. However, this could not be observed experimentally for two reasons. First, as other decoherence mechanisms limit the hyperfine T_2 to ≤ 1 s [Rancic et al., 2018]; second, the magnetic field noise in our experiment will likely contain several frequency components: A dominant base frequency of 1.4 Hz originating from the cryocooler cycle, some higher harmonics, and potentially other frequencies in the Hz range originating from mechanical resonances of the setup. These different frequency contributions would lead to revivals at different times, dampening the amplitude of both partial and full revivals. A thorough quantification of these effects is beyond the scope of our manuscript.

3d) It would be helpful if the authors were consistent in denoting the sequence duration. Both T_S and 2\tau are used. I don't think T_S is defined.

To clarify, we added a definition of τ and T_S at the beginning of the Hahn-echo section. Additionally, we changed the axis label of Fig. S19 to T_S, to match the axis label in Fig. S18.

3e) In the discussion, the authors rightfully assert that other noise mechanisms might be present, and that classical magnetic field noise cannot explain all experimental findings such as the varying stretching factor across different transitions. I would like to see this reservation reflected in the main text, which confidently pinpoints magnetic field noise as the sole cause. Adding one or two words should be enough to qualify the claims (the details are indeed more appropriate for the supplementary information).

In the discussion section, we now write "*primary limitation*" instead of "*current limitation*", suggesting that there are secondary effects that are addressed in the supplementary information.

In addition, we modified the nuclear-spin qubit coherence section: "*This reduction, and the revival of T_{Hahn} around 30 ms, are attributed to fluctuations of the bias magnetic field*".

4) The supplementary information on Raman control states, "Thus, we implement coherent control via optical Raman fields, detuned by ≈ 0.9 GHz". To me, this implies a one-photon detuning, yet the meaning must be that the two field components are split by 0.9 GHz in accordance with the ground state splitting.

We agree with the referee that this sentence can be misunderstood. Indeed, we refer to the frequency splitting here and not the one-photon detuning. To clarify, we adapted the text: "*we implement coherent control via optical Raman fields whose frequency difference of 0.9 GHz matches the ground-state level splitting*"

5) In the supplementary section on Raman pulse fidelity, the authors state "The damping constant Td hereby corresponds to the lower bound of the emitter dephasing time T_2^*." This statement is wrong. Driving a qubit protects it against dephasing from T_2*. In the limit of Omega tending to infinity, the grand Rabi frequency no longer depends on the two-photon detuning, and the oscillations continue forever. A nice experimental example of protecting a solid-state spin through driving can be found in [Bodey, J.H., Stockill, R., Denning, E.V. et al. Optical spin locking of a solid-state qubit. npj Quantum Inf 5, 95 (2019).]
We thank the referee for pointing out that this sentence was inaccurate, as driving the qubit can indeed protect it against dephasing. We have modified the sentence accordingly: *We find the longest damping constant T_d =2.4(3) ms when the emitter is driven at the lowest Rabi frequency [...].* In addition, we modified the caption of Fig. S17 accordingly: *"Rabi oscillations and hyperfine dephasing of an ^{167}Er dopant".*

I also agree with the other reviewer that the presentation is lengthy and could be made more concise by cutting down on repetitions. A few examples:
1. Too much information is repeated in the figure captions. This is especially true for the Fig. 1a caption, which repeats the motivation of the large magnetic field rather than concisely describing the elements of the figure.

We believe that the information about the effect of a large B-field is required to understand the meaning of the symbols in the figure. Therefore, we prefer to keep the level of detail in the mentioned figure caption.

2. Similarly, the concept and limitations of qubit readout through photon counting is verbosely explained in both the main text and the caption of Fig. 3. Given how well established this technique is, the treatment could be shortened.

To make our work easily accessible to a broad audience, as targeted by Nature Communications, we would prefer to keep the caption in the submitted form. We believe that the technique may not be known to readers outside of the field, and thus a very brief explanation helps to understand the content of the figure.

3. The textbook theory treatment of Raman control in the supplementary information is not necessary. It is sufficient to quote the main results for a Lambda system by providing a reference. I find it odd that the authors include atomic recoil in the two-photon detuning (Supplementary equation 19) when it clearly doesn't apply to the studied system. Please don't add irrelevant information.

While many readers with a background in atomic physics will be familiar with Raman control, it may be less known to readers working on solid-state emitters, where the technique is less common. We therefore prefer to keep the short introduction, as it provides the basis for the discussion of the impact of cavity frequency fluctuations. To avoid unnecessary information, however, we removed the recoil term in equation 19, instead of explaining why it can be dropped.

These suggestions are not crucial towards accepting the paper, but would, in my opinion, greatly improve the readability.
While the manuscript has been improved, I cannot recommend publication until my objections on the Hahn Echo analysis have been addressed.
We thank the referee for their careful reading and the suggestions that led to further improvements of the manuscript. With the added clarifications regarding the echo analysis, we hope that publication can now be recommended without reservations.

Reviewer #2 (Remarks to the Author):

I am satisfied with the revised manuscript and recommend publication

Reviewer #3 (Remarks to the Author):

I co-reviewed this manuscript with one of the reviewers who provided the listed reports. This is part of the Nature Communications initiative to facilitate training in peer review and to provide appropriate recognition for Early Career Researchers who co-review manuscripts.
